# Exact model reduction of combinatorial reaction networks

**DOI:** 10.1186/1752-0509-2-78

**Published:** 2008-08-28

**Authors:** Holger Conzelmann, Dirk Fey, Ernst D Gilles

**Affiliations:** 1 Max-Planck Institute for Dynamics of Complex Technical Systems, Sandtorstr. 1, 39106, Magdeburg, Germany; 2 Industrial Control Centre, Department of Electronic & Electrical Engineering, University of Strathclyde, Glasgow, Scotland, UK

## Abstract

**Background:**

Receptors and scaffold proteins usually possess a high number of distinct binding domains inducing the formation of large multiprotein signaling complexes. Due to combinatorial reasons the number of distinguishable species grows exponentially with the number of binding domains and can easily reach several millions. Even by including only a limited number of components and binding domains the resulting models are very large and hardly manageable. A novel model reduction technique allows the significant reduction and modularization of these models.

**Results:**

We introduce methods that extend and complete the already introduced approach. For instance, we provide techniques to handle the formation of multi-scaffold complexes as well as receptor dimerization. Furthermore, we discuss a new modeling approach that allows the direct generation of exactly reduced model structures. The developed methods are used to reduce a model of EGF and insulin receptor crosstalk comprising 5,182 ordinary differential equations (ODEs) to a model with 87 ODEs.

**Conclusion:**

The methods, presented in this contribution, significantly enhance the available methods to exactly reduce models of combinatorial reaction networks.

## Background

A large problem in mathematical modeling of biochemical signal transduction networks is combinatorial complexity [1]. Due to the occurrence of receptors and scaffold proteins with high numbers of binding domains and binding partners the number of feasible molecular species is enormous [1-5]. Many existing models evade the problem of combinatorial variety by assuming reduced, heuristic network structures that focus on a restricted number of molecular species and reactions [6-14]. Faeder *et al*. [15] showed by simulation studies that in combinatorial reaction networks only a relatively small part of the network might be active which means that the concentration of many species is negligible low. By eliminating these species as well as the associated reactions a fairly reduced model can be obtained. Faeder *et al*. [15] also showed that the predictions of such a reduced model match those of the complete one for the original parameter values quite well, but that the reduced model is not predictive over a larger range of parameter values. Even very small perturbations in the parameters may result in large approximation errors. Since the real kinetic parameters are usually unknown, these findings of Faeder *et al*. [15] indicate that a heuristically derived model structure will mostly be insufficient to approximate the dynamics of the real signaling network. This result is confirmed by Conzelmann *et al*. [16] who showed, by discussing a scaffold protein with only three binding domains, that reasonable, heuristically derived, model reductions may lead to significant approximation errors even for very small reaction networks.

An alternative approach to tackle combinatorial complexity is stochastic simulation. One possibility is an agent-based approach in which each protein is considered to be an autonomous individual in the reaction network. This approach restricts the number of elements that have to be considered to the total number of protein copies, while the number of feasible multiprotein complexes, which can easily grow to billions [1,3], often exceeds this number by far. However, the computational cost for that kind of stochastic simulations can also be extremely high. Another possibility how stochastic models can help to reduce combinatorial complexity has been presented by Lok *et al*. [17]. The enormous complexity of the stochastic model is reduced by a new approach which incorporates complexes and reactions only when they are needed as the simulation proceeds [17,18]. However, it is much harder to analyze the dynamic behavior of stochastic models or to identify the model parameters from measurements [18].

Another, ODE based, approach has been introduced by Blinov *et al*. [19]. The modeling tool BIONETGEN allows a rule-based model specification, which is automatically expanded to a complete mechanistic ODE model. BIONETGEN has been used to create a number of signaling models including EGF receptor signaling and Fc*ϵ*RI signaling [20,21].

Borisov *et al*. [22,23] proposed an alternative approach which adopts the point of view that the fundamental elements of signal transduction are domains instead of molecular species [24]. Borisov *et al*. suggested to substitute the common mechanistic network description that includes all individual molecular species by a macrodescription. In this context, the term *micro-state *is used to describe individual multiprotein complexes, whereas the term *macro-state *refers to large sets of micro-states sharing a certain characteristic like phosphorylation of a defined binding domain. These macro-states correspond to descriptive biological quantities like phosphorylation degrees or levels of occupancy. The goal of this approach is the generation of a dynamic ODE model describing the concentrations of these macro-states. Borisov *et al*. [22] show for instance that in the case of independent binding domains a reduced number of ODEs is sufficient to describe the dynamics of the macro-states accurately. This approach has been extended by Conzelmann *et al*. [16] who introduced a systematic procedural method. The starting point of this method is a complete mechanistic ODE model which is subsequently reduced [16]. The reduction method bases on a hierarchically structured state space transformation. However, Conzelmann *et al*. [16] only provide a general transformation pattern for scaffold proteins with numerous single protein ligands. If these ligands can recruit further signaling proteins or the considered scaffold forms a dimer, the transformation pattern has to be extended. In this contribution, we present these extensions that enhance the applicability of the method to all kinds of signaling networks. Furthermore, we introduce a novel approach to directly generate the reduced equations. This approach circumvents the computationally expensive set-up of a full combinatorial model and its subsequent reduction.

The Section *Mathematical Prerequisites *will briefly discuss the mathematical concepts that are used. The main results are discussed in three parts: Section *Exact Model Reduction *presents the extension of the transformation pattern introduced by Conzelmann *et al*. [16]. Section *Reduced Order Modeling of Combinatorial Reaction Networks *introduces the novel approach for directly generating reduced models. Section *Example: EGF and Insulin Receptor Crosstalk *discusses the methods practical applicability and its benefits by generating a fairly reduced model of EGF and insulin receptor crosstalk.

## Methods

### Mathematical prerequisites

In this contribution, exact model reductions will be discussed, but the term *exact *may be misleading since the elimination of model equations is always linked to loss of information. A reduction can only be exact in terms of the input/output behavior of the system which can be exactly preserved by a reduced model. In this work, we will always consider ODE models given in state space representation

(1) $$\begin{array}{l} \begin{array}{cc} \dot{\vec{x}}(t)=\vec{f}(\vec{x}(t),\vec{u}(t)) & \vec{x}(t_{0})={\vec{x}}_{0} \end{array}\\ \vec{y}(t)=\vec{h}(\vec{x}(t)), \end{array}$$

where $\vec{x}$(*t*) denotes the *n*-dimensional vector of all dynamic states or variables and $\vec{u}$(*t*) represents the *m*-dimensional vector of all external input signals. The vector $\vec{y}$(*t*) comprises all *q *output variables of the system, which either correspond to measured quantities or more general to all quantities of interest. The vector field $\vec{f}$ and the vector valued function $\vec{h}$ do have appropriate dimensions.

Since we assume that the output $\vec{y}$ does include all essential quantities, we are solely interested in the systems input/output behavior. It is very essential to stress that exact reducibility is solely determined by the system's structure and the definition of input and output variables. Thus, the only way to influence exact reducibility is to modify a system or to change the input and output variables. However, in most cases neither the model structure nor the choice of input and output variables is discretionary. Exact reduction methods should not try to influence exact reducibility of a model but provide the information whether a given system is exactly reducible and how it can be reduced. The question of whether a model is exactly reducible is closely related to the control theoretical concepts of observability and controllability which will be introduced below. In control theory models that are not exactly reducible are called *minimal realizations *[25]. If a model is no minimal realization it comprises uncontrollable or unobservable states, which can be eliminated without changing the systems input/output behavior. The elimination of unobservable and uncontrollable states can be achieved by a formal dissection of the model's state space into observable and controllable, observable but not controllable, controllable but not observable as well as neither observable nor controllable subsystems. Such a dissection is called *Kalman decomposition *[26].

Control theory provides a couple of methods that facilitate the separation of controllable and uncontrollable as well as of observable and unobservable states. However, a deficiency of these methods is that they are either developed for linear systems or they are only designed for small nonlinear models [25], but they are not applicable to very large nonlinear models of biological signaling cascades.

The methods we developed and present facilitate a Kalman decomposition of combinatorial reaction networks. Interestingly, for the considered type of systems a Kalman decomposition can be achieved by a linear state space transformation that is additionally independent of the systems parametrization or the choice of input and output variables. Naturally, a change of the parameter values or the input and output variables can affect the number of observable and controllable states but the proposed transformation always provides a Kalman decomposition. This statement can be verified using the approach discussed in the Section *Generality of the Method*.

### Mathematical characterization of processes and process interactions

A crucial problem in modeling combinatorial reaction networks is the parametrization of the immense number of reactions. Considering different signal transduction models [10,12,20,27] and modeling techniques [12,19,22,16,3] it becomes apparent that mostly a large number of occurring reactions is parametrized by a relatively small number of distinct kinetic parameters. However, the assumptions, which reactions are parametrized by the same, and which by distinct parameter values, differ. From our point of view the most suggestive assumption is to determine parametrization on the basis of process interactions. Our focus is on domains of large scaffold or receptor proteins that can be either occupied by other proteins or can undergo post-translational modifications like phosphorylation. We define a binding process as the sum of all reactions that change the level of occupancy of a considered domain. Analogously, we define a modification process as the sum of all reactions changing the degree of modification of a domain. Two arbitrary processes, no matter if binding or modification processes, may be either completely independent, interact unidirectionally or mutually. Koschorreck *et al*. [3] additionally discuss so-called all-or-none interactions which represent an important border case of mutually interacting processes. These different types of interactions shall be exemplified considering a very simple example which is taken from [28]. In this example, a receptor *R *is considered, which recruits the two ligands *L *and *E*. Hence, the system comprises two binding processes. In this case, the reaction system consists of four reversible reactions, for which the following reaction rates can be formulated using mass action kinetics

(2) $$\begin{array}{l} r_{1}=k_{1}[R(0,0)]\cdot [L]-k_{-1}[R(L,0)]\\ r_{2}=k_{2}[R(0,E)]\cdot [L]-k_{-2}[R(L,E)]\\ r_{3}=k_{3}[R(0,0)]\cdot [E]-k_{-3}[R(0,E)]\\ r_{4}=k_{4}[R(L,0)]\cdot [E]-k_{-4}[R(L,E)]. \end{array}$$

According to Conzelmann *et al*. [28] the following process interaction types can be distinguished

• **non-interacting processes**

Complete independence implies that the kinetic association and dissociation constants of one domain do not change upon ligand binding on the other domain. Hence, it follows for the parameters *k*_2 _= *k*_1_, *k*_-2 _= *k*_-1_, *k*_4 _= *k*_3 _and *k*_-4 _= *k*_-3_.

• **unidirectionally interacting processes**

The binding of one ligand, e.g. ligand *L*, is not influenced by binding of the other one. However, *L *binding does change the kinetic properties of the other domain. In this case, only the conditions *k*_2 _= *k*_1 _and *k*_-2 _= *k*_-1 _have to be fulfilled.

• **mutually interacting processes**

This is the most general case. Binding of a ligand has an influence on binding of the other ligand and vice versa. In this case all parameters can have different values.

In addition to these, Koschorreck *et al*. [3] introduce

• **all-or-none interactions**

All-or-none interactions are a special case of mutual interactions. An mutual interaction between two processes is called all-or-none interaction, if the reaction cycle given by the four reactions of our example degenerates to a reaction chain. In real biochemical networks such interactions usually are given by domain phosphorylation and subsequent ligand binding. Mostly phosphorylation can be considered as necessary precondition for ligand binding. On the other side ligand binding prevents dephosphorylation of the domain for steric reasons. To realize an all-or-none interaction in our example, one has to choose *k*_2 _= *k*_-2 _= *k*_3 _= *k*_-3 _= 0. In this case the species *R*(0, *E*) will not occur and the remaining reactions *r*_1 _and *r*_4 _form a reaction chain.

As can be seen from the examples provided below as well as from examples discussed in other publications [22,23,16,3], the absence of interactions or the occurrence of unidirectional and all-or-none interactions facilitate model reduction and modularization.

### State space transformations

The model reduction approach presented by Conzelmann *et al*. [16] is based on a linear state space transformation

(3) $$\vec{z}=T\vec{x}.$$

In this work, it is assumed that each transformation matrix *T *is an invertible square matrix with real values [25]. The transformed model equations can be deduced from Equation 3 by differentiation

(4) $$\begin{array}{ccc} \vec{z}=T\vec{x} & |\frac{d}{dt} & \begin{array}{cc} \Rightarrow & \dot{\vec{z}}=T\dot{\vec{x}}=T\vec{f}(\vec{x},\vec{u}) \end{array} \end{array}.$$

Finally, the old variables $\vec{x}$ have to be replaced by the new ones using the inverse transformation $\vec{x}=T^{-1}\vec{z}$ resulting in

(5) $$\begin{array}{l} \begin{array}{ll} \dot{\vec{z}}(t)=T\vec{f}(T^{-1}\vec{z}(t),\vec{u}(t)) & \vec{z}(t_{0})={\vec{z}}_{0}=T{\vec{x}}_{0} \end{array}\\ \vec{y}(t)=\vec{h}(T^{-1}\vec{z}(t)). \end{array}$$

In the case of nonlinear transformations, the transformed model equations can be deduced following the same procedure. However, note that the inversion of a nonlinear transformation can be extremely difficult.

### Observability

Let us consider the linear ODE system

(6) $$\begin{array}{c} \left[\begin{array}{c} {\dot{\vec{x}}}_{1}\\ {\dot{\vec{x}}}_{2} \end{array}\right]=\left[\begin{array}{cc} A_{1,1} & 0\\ A_{2,1} & A_{2,2} \end{array}\right]\left[\begin{array}{c} {\vec{x}}_{1}\\ {\vec{x}}_{2} \end{array}\right]+\left[\begin{array}{c} B_{1}\\ B_{2} \end{array}\right]\vec{u}\\ \vec{y}=C_{1}{\vec{x}}_{1}, \end{array}$$

in which *A*, *B *and *C *are constant matrices of appropriate dimensions and the initial conditions are given by ${\left[{\vec{x}}_{1}(0),{\vec{x}}_{2}(0)\right]}^{T}={\left[{\vec{x}}_{1,0},{\vec{x}}_{2,0}\right]}^{T}$. Obviously, the variables denoted as ${\vec{x}}_{2}$ do not have any influence on the output variables $\vec{y}$. Hence, any initial states whose values for ${\vec{x}}_{1,0}$ coincide result in identical outputs for arbitrary initial conditions ${\vec{x}}_{2,0}$. The differences in the states ${\vec{x}}_{2}$ cannot be observed considering these outputs.

The number of observable states is determined by the dimension of the so-called observability space. For linear systems it can be calculated as

(7) *d *= rank(*Q*)   with   *Q *= [*C*, *C A*, . . . *C A*^*n*-1^]^*T*^

The first *d *linearly independent rows of *Q *build a basis for the observability space O[25]. For *d *= *n *the system is called observable.

These considerations imply that an unobservable system always can be reduced without affecting the dynamics of the output variables. In Equation 6 a reduced system would exclusively comprise the ODEs for the state variables ${\vec{x}}_{1}$. Such reductions are exact with respect to the output.

### Controllability

Now let us consider a differently structured linear system of the form

(8) $$\begin{array}{cc} \left[\begin{array}{c} {\dot{\vec{x}}}_{1}\\ {\dot{\vec{x}}}_{2} \end{array}\right]=\left[\begin{array}{cc} A_{1,1} & A_{1,2}\\ 0 & A_{2,2} \end{array}\right]\left[\begin{array}{c} {\vec{x}}_{1}\\ {\vec{x}}_{2} \end{array}\right]+\left[\begin{array}{c} B_{1}\\ 0 \end{array}\right]\vec{u} & \vec{x}(0)={\vec{x}}_{0}\\ \vec{y}=C\vec{x}. & \end{array}$$

In this case the state variables ${\vec{x}}_{2}$ cannot be influenced by the inputs. Hence, the chosen input does not allow to control the system in any desired way, why the system is said to be *uncontrollable *[25]. In analogy to the considerations about observability, there also exists a controllability space C whose dimension as well as its basis can be deduced from the matrix

(9) *P *= [*B A B *. . . *A*^*n*-1^*B*]

Uncontrollable systems also can be reduced without affecting the dynamics of the output, if an additional assumption is fulfilled. If the dynamics of the uncontrollable subsystem (${\vec{x}}_{2}$ in Example 8) already decayed, ${\vec{x}}_{2}$ can be replaced by its steady state value which in the regarded example corresponds to zero.

## Results and discussion

### Exact model reduction

#### Short review

Conzelmann *et al*. have introduced linear transformations that realize a Kalman decomposition for models of scaffold proteins and receptors with single protein ligands [16]. The term *single protein ligand *indicates that one only considers the multi domain protein and its direct binding partners but no additional binding or modification processes at these ligands.

The model reduction procedure suggested by Conzelmann *et al*. [16] can be divided into three essential steps.

**Step 1: **One generates a complete mechanistic ODE model of the considered combinatorial reaction network using e.g. BIONETGEN or ALC [19,29]. Furthermore, one has to define input and output variables.

**Step 2: **The ODE model is transformed using the proposed linear transformation pattern. If the system contains unobservable state variables the transformed model equations can be written as

(10) $$\begin{array}{c} \left[\begin{array}{c} {\dot{\vec{z}}}_{1}\\ {\dot{\vec{z}}}_{2} \end{array}\right]=\left[\begin{array}{c} {\vec{g}}_{1}({\vec{z}}_{1},\vec{u})\\ {\vec{g}}_{2}({\vec{z}}_{1},{\vec{z}}_{2},\vec{u}) \end{array}\right]\\ \vec{y}=\vec{h}({\vec{z}}_{1}). \end{array}$$

In analogy to Equation 6 the states ${\vec{z}}_{2}$ are unobservable. Choosing an invertible transformation matrix assures that the system's dynamics are preserved, and the original states can be retrieved from the new ones at any time as long as none of the transformed equations are eliminated.

**Figure 1:**
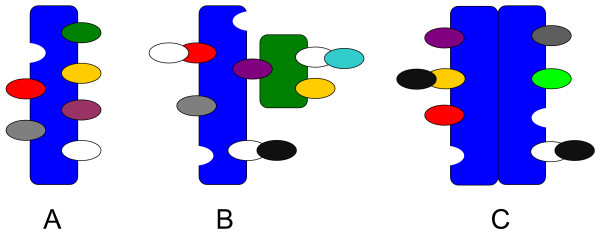
The three basic scenarios that will be discussed in the following. Figure A depicts a receptor or scaffold protein with single protein ligands, i.e. each binding domain can recruit single proteins which do not possess further binding domains. A scaffold with multiprotein ligands is shown in Figure B. Some of the ligands are scaffolds themselves. The last scenario additionally includes receptor homodimerization. Heterodimerization on the other site corresponds to the scenario depicted in Figure B.

**Step 3: **The last reduction step is the elimination of the unobservable system states. If the model also comprises uncontrollable states these ODEs can be replaced by the related steady state equations. A suitable transformation pattern that facilitates a Kalman decomposition of models describing scaffolds with multiprotein ligands or scaffold homodimerization is still missing. The term *multprotein ligand *indicates that the direct binding partners of the considered scaffold can also recruit further proteins or scaffolds (see Figure 1). In the following subsections, we will introduce and discuss transformation patterns for these kind of systems.

#### Scaffolds with multiprotein ligands

Many scaffold proteins or receptors often recruit other scaffolds which in turn can be phosphorylated and/or bind further ligands. A prominent example is the scaffold IRS which binds to insulin receptor and can recruit numerous other ligands like Grb2 or PI3K [30]. We will call scaffolds like IRS multiprotein ligands. Note that, in general, these multiprotein ligands can either bind single protein ligands or again other multiprotein ligands. Heterodimerization, as it occurs in the ErbB signaling network [31], also fits into this pattern. Homodimerization, on the other hand, will be excluded from the following considerations. Due to the symmetry of homodimeric complexes homodimerization has to be handled differently as will be discussed later. Scaffolds with single proteins ligands as discussed by Conzelmann *et al*. [16] can be considered as a special case of what we examine here. The main difference between these multiprotein and single protein ligand systems is the formation of long protein chains. For this reason, we will focus on this phenomenon and its mathematical treatment. Later we will exemplify how branched multiprotein ligand systems can be handled considering a model of EGF and insulin receptor crosstalk.

Let us focus on a receptor protein *R *which provides *n *binding domains. We take the assumption that each domain *i *can bind an effector protein $E_{1}^{i}$ which in turn can recruit another effector protein $E_{2}^{i}$ till finally $E_{m-1}^{i}$ binds $E_{m}^{i}$. In order to reduce the number of indices we also presume that each chain of effector proteins consists of *m *proteins. Finally, we only regard binding processes and neglect all domain phosphorylations. Thus, each receptor domain can be either unoccupied or occupied by a multiprotein ligand consisting of one to *m *proteins which results in (*m *+ 1)^*n *^distinct receptor complexes. Furthermore, the *m *effectors that form the different multiprotein ligands for one single receptor domain can build $\frac{m(m+1)}{2}$ distinct complexes. According to these examinations the total number of feasible multiprotein species is ${\left(1+m\right)}^{n}+\frac{m(m+1)}{2}n$.

##### General Transformation Pattern

In analogy to the considerations made by Conzelmann *et al*. [16] we require a state space transformation which facilitates a Kalman decomposition of the reaction network. The transformation pattern introduced by Conzelmann *et al*. [16] can be divided into tiers that describe levels of occupency of different order. The concept of using levels of occupancy as new variables is problematic for the considered multiprotein ligand systems. The term *level of occupancy *implies a certain hierarchy among the signaling proteins, which is certainly given in the single protein ligand scenario where one scaffold can bind numerous other effector proteins. It is obvious that in such reaction networks the scaffold takes up a prominent position which suggests the consideration of its occupancy levels. In a system that involves numerous scaffolds a clear hierarchy is missing, and the question arises which occupancy levels should be considered. In most cases, an intuitive hierarchy will be automatically chosen. For example, in the case of insulin signaling, it is quite suggestive to choose the insulin receptor as central protein of the cascade. Due to representational reasons we also assume a hierarchy in our examples with *R *being the central protein. However, if one e.g. considers heterodimerization of two ErbB receptors [31] it is not apparent which receptor takes up a more prominent position than the other one. Another problem is that the definition of occupancy levels for multiprotein ligand systems is not as unique as for single protein ligand systems. The quantity [*R*($E_{1}^{1}$, *, ..., *)], which can be interpreted as an occupancy level, describes all receptor species whose first domain is occupied by the single protein $E_{1}^{1}$ excluding all species in which $E_{1}^{1}$ has bound any further effectors or scaffolds. [*R*($E_{1}^{1}$(*), *, ..., *)] on the other site represents an alternative type of occupancy level which does not exclude the previously mentioned multiprotein complexes.

A more general transformation pattern is required that avoids the implication of molecular hierarchy, and is consistent with the transformation pattern introduced by Conzelmann *et al*. [16]. These requirements are met by the introduction of so-called *occurrence levels*. Occurrence levels always refer to a certain molecular subcomplex and correspond to the sums of all multiprotein species that comprise this subcomplex. Thus, for each individual molecular species one can define a respective occurrence level. If these occurrence levels are used to replace the original model states, this defines a linear and invertible transformation. The proposed transformation pattern also preserves the hierarchical structure of the transformation matrix. The 0^th ^tier of the transformation matrix, as introduced by Conzelmann *et al*. [16], includes the overall concentrations of all involved signaling proteins. It corresponds to the occurrence levels of individual proteins which are a very special type of subcomplex. The 1^st ^tier includes the occurrence levels of all possible two protein subcomplexes. In the case of scaffolds with single protein ligands this directly corresponds to the levels of occupancy. According to this pattern the following tiers of the transformation matrix respectively comprise all subcomplexes consisting of three, four and more proteins. If phosphorylations occur in the considered reaction network the phosphate groups have to be treated as additional molecules. For example a scaffold with one phosphorylated domain is regarded as a two molecule complex.

For the simplified case introduced above the new transformed states can be specified as follows. The 0^th ^tier comprises the states [*R*(*, ..., *)] and [$E_{j}^{i}$(*)], while the first one includes the states [*R*(*, ..., *, $E_{1}^{i}$(*), *, ..., *)] as well as $\left[E_{j}^{i}(E_{j+1}^{i}(*))\right]$. The occurrence levels that refer to all three molecule complexes are [*R*(*, ..., *, $E_{1}^{i}$(*), *, ..., *, $E_{1}^{k}$(*), *, ..., *)], [*R*(*, ..., *, $E_{2}^{i}$(*), *, ..., *)] and $\left[E_{j}^{i}(E_{j+2}^{i}(*))\right]$. The subsequent tiers are defined according to this pattern, and the last tier only comprises the single micro-state $\left[R(E_{m}^{1},...,E_{m}^{n})\right]$. The fact that each individual molecular species can be uniquely linked to an associated occurrence level suggests that the transformation is invertible. This can also be proven by using the mathematical induction as described for single protein ligands in Conzelmann *et al*. [16] (data not shown).

#### Examples

**Figure 2:**
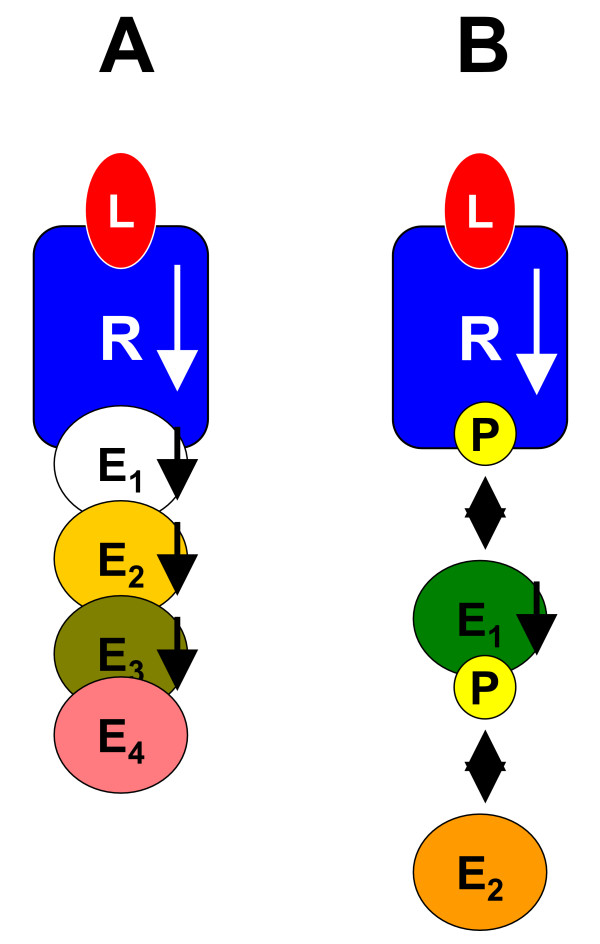
Examples for multiprotein ligand systems. Figure A depicts a chain of signaling proteins without any post-translational modifications such as phosphorylations. All bindings are assumed to interact unidirectionally with each other (black unidirectional arrows). Figure B shows a similar system including domain phosphorylation. Thereby, it is assumed that phosphorylation and subsequent effector binding interact via an all-or-none reaction. Since all-or-none interactions are always bidirectional they are depicted by bidirectional arrows. The last example is a small part of the insulin signaling pathway.

We will analyze two different systems of receptors with multiprotein ligands (see Figure 2). For the sake of simplicity these examples solely consider chains of signaling proteins. A more complex example is given in the Section *Example: EGF and Insulin Receptor Crosstalk*. The first system consists of six signaling proteins which bind consecutively to each other. In order to provide a simple representation of the occurring complexes and the corresponding occurrence levels the protein *R *is considered as central receptor which binds the single protein ligand *L *and a multiprotein ligand consisting of the effectors *E*_1 _to *E*_4_. None of these proteins is assumed to be phosphorylated. The second example only comprises four signaling proteins of which three are phosphorylated.

##### Example 1: Six signaling proteins

We assume that *E*_1 _binding is unidirectionally influenced by the recruitment of *L*. Equivalently, *E*_*i *_binding is unidirectionally influenced by the binding of *E*_*i*-1 _to its predecessor. The resulting reaction rules for this system are given in Table 1. We take the assumption that [*L*] is the input of the system. The choice of output variables is more difficult in this example. For systems with single protein ligands the levels of occupancy are chosen. These correspond to all states included in the 1^st ^transformation tier. In accordance to this choice one could again take all states of the 1^st ^tier as output variables. For multiprotein ligands these states correspond to the occurrence levels of all two protein complexes. However, for many real networks also other states might be of interest. Let us consider the insulin receptor which can recruit a multiprotein ligand consisting of Shc, Grb2 and SOS [30,32]. In this case, the recruitment of SOS to a membrane bound signaling complex initiates the MAPK cascade [32]. Thus, it is important to know how many SOS proteins have bound to the receptor complex and not how many Grb2-SOS complexes occur in the network. Hence, we will consider two types of output variables.

**Table 1:** Reaction rules describing the Example depicted in Figure 2A.

*R*(0, *)	+	*L *	⇋	*R*(*L*, *)	*k*_1_, *k*_-1_
*R*(0, 0)	+	*E*_1_(*)	⇋	*R*(0, *E*_1_(*))	*k*_2_, *k*_-2_
*R*(*L*, 0)	+	*E*_1_(*)	⇋	*R*(*L*, *E*_1_(*))	*k*_3_, *k*_-3_
*E*_1_(0, 0)	+	*E*_2_(*)	⇋	*E*_1_(*E*_2_(*))	*k*_4_, *k*_-4_
*R*(*, *E*_1_)	+	*E*_2_(*)	⇋	*R*(*, *E*_1_(*E*_2_(*)))	*k*_5_, *k*_-5_
*E*_2_(0, 0)	+	*E*_3_(*)	⇋	*E*_2_(*E*_3_(*))	*k*_6_, *k*_-6_
*E*_1_(*, *E*_2_)	+	*E*_3_(*)	⇋	*E*_1_(*, *E*_3_(*))	*k*_7_, *k*_-7_
*E*_3_(0, 0)	+	*E*_4_	⇋	*E*_3_(*E*_4_)	*k*_8_, *k*_-8_
*E*_2_(*, *E*_3_)	+	*E*_4_	⇋	*E*_2_(*, *E*_4_)	*k*_9_, *k*_-9_

The kinetic parameters are specified behind the rules.

The output vector ${\vec{y}}_{1}$ represents the five occurrence levels of the 1^st ^transformation tier. The output vector ${\vec{y}}_{2}$ on the other hand includes the variables [*R*(*L*, *)], [*R*(*, *E*_1_(*))], [*R*(*, *E*_2_(*))], [*R*(*, *E*_3_(*))] and [*R*(*, *E*_4_)]. In order to show the large influence of process interactions on exact model reduction we additionally consider the case that *k*_8 _= *k*_9 _and *k*_-8 _= *k*_-9_. This assumption implies that *E*_4 _binding is completely independent of all other binding processes. Since the transformation pattern is independent of the kinetic system properties and the chosen output variables all mentioned cases can be handled using the same transformation. It consists of six tiers that are shown in Table 2.

**Table 2:** Hierarchical transformation that realizes a Kalman decomposition for the example system depicted in Figure 2A.

[*R*(*, *)]	=	[*R*(0, 0)] + [*R*(0, *E*_1_)] + [*R*(0, *E*_2_)] + [*R*(0, *E*_3_)] + [*R*(0, *E*_4_)] + [*R*(*L*, 0)] + [*R*(*L*, *E*_1_)] + [*R*(*L*, *E*_2_)] + [*R*(*L*, *E*_3_)] + [*R*(*L*, *E*_4_)]
[*E*_1_(*)]	=	[*E*_1_(0)] + [*E*_1_(*E*_2_)] + [*E*_1_(*E*_3_)] + [*E*_1_(*E*_4_)] + [*R*(0, *E*_1_)] + [*R*(0, *E*_2_)] + [*R*(0, *E*_3_)] + [*R*(0, *E*_4_)] + [*R*(*L*, *E*_1_)] + [*R*(*L*, *E*_2_)] + [*R*(*L*, *E*_3_)] + [*R*(*L*, *E*_4_)]
[*E*_2_(*)]	=	[*E*_1_(*E*_2_)] + [*E*_1_(*E*_3_)] + [*E*_1_(*E*_4_)] + [*E*_2_(0)] + [*E*_2_(*E*_3_)] + [*E*_2_(*E*_4_)] + [*R*(0, *E*_2_)] + [*R*(0, *E*_3_)] + [*R*(0, *E*_4_)] + [*R*(*L*, *E*_2_)] + [*R*(*L*, *E*_3_)] + [*R*(*L*, *E*_4_)]
[*E*_3_(*)]	=	[*E*_1_(*E*_3_)] + [*E*_1_(*E*_4_)] + [*E*_2_(*E*_3_)] + [*E*_2_(*E*_4_)] + [*E*_3_(0)] + [*E*_3_(*E*_4_)] + [*R*(0, *E*_3_)] + [*R*(0, *E*_4_)] + [*R*(*L*, *E*_3_)] + [*R*(*L*, *E*_4_)]
[*E*_4_(*)]	=	[*E*_1_(*E*_4_)] + [*E*_2_(*E*_4_)] + [*E*_3_(*E*_4_)] + [*E*_4_(0)] + [*R*(0, *E*_4_)] + [*R*(*L*, *E*_4_)]
		
[*R*(*L*, *)]	=	[*R*(*L*, 0)] + [*R*(*L*, *E*_1_)] + [*R*(*L*, *E*_2_)] + [*R*(*L*, *E*_3_)] + [*R*(*L*, *E*_4_)]
[*R*(*, *E*_1_(*))]	=	[*R*(0, *E*_1_)] + [*R*(0, *E*_2_)] + [*R*(0, *E*_3_)] + [*R*(0, *E*_4_)] + [*R*(*L*, *E*_1_)] + [*R*(*L*, *E*_2_)] + [*R*(*L*, *E*_3_)] + [*R*(*L*, *E*_4_)]
[*E*_1_(*E*_2_(*)]	=	[*E*_1_(*E*_2_)] + [*E*_1_(*E*_3_)] + [*E*_1_(*E*_4_)] + [*R*(0, *E*_2_)] + [*R*(0, *E*_3_)] + [*R*(0, *E*_4_)] + [*R*(*L*, *E*_2_)] + [*R*(*L*, *E*_3_)] + [*R*(*L*, *E*_4_)]
[*E*_2_(*E*_3_(*))]	=	[*E*_1_(*E*_3_)] + [*E*_1_(*E*_4_)] + [*E*_2_(*E*_3_)] + [*E*_2_(*E*_4_)] + [*R*(0, *E*_3_)] + [*R*(0, *E*_4_)] + [*R*(*L*, *E*_3_)] + [*R*(*L*, *E*_4_)]
[*E*_3_(*E*_4_(*))]	=	[*E*_1_(*E*_4_)] + [*E*_2_(*E*_4_)] + [*E*_3_(*E*_4_)] + [*R*(0, *E*_4_)] + [*R*(*L*, *E*_4_)]
		
[*R*(*L*, *E*_1_(*))]	=	[*R*(*L*, *E*_1_)] + [*R*(*L*, *E*_2_)] + [*R*(*L*, *E*_3_)] + [*R*(*L*, *E*_4_)]
[*R*(*, *E*_2_(*))]	=	[*R*(0, *E*_2_)] + [*R*(0, *E*_3_)] + [*R*(0, *E*_4_)] + [*R*(*L*, *E*_2_)] + [*R*(*L*, *E*_3_)] + [*R*(*L*, *E*_4_)]
[*E*_1_(*E*_3_(*)]	=	[*E*_1_(*E*_3_)] + [*E*_1_(*E*_4_)] + [*R*(0, *E*_3_)] + [*R*(0, *E*_4_)] + [*R*(*L*, *E*_3_)] + [*R*(*L*, *E*_4_)]
[*E*_2_(*E*_4_(*))]	=	[*E*_1_(*E*_4_)] + [*E*_2_(*E*_4_)] + [*R*(0, *E*_4_)] + [*R*(*L*, *E*_4_)]
		
[*R*(*L*, *E*_2_(*))]	=	[*R*(*L*, *E*_2_)] + [*R*(*L*, *E*_3_)] + [*R*(*L*, *E*_4_)]
[*R*(*, *E*_3_(*))]	=	[*R*(0, *E*_3_)] + [*R*(0, *E*_4_)] + [*R*(*L*, *E*_3_)] + [*R*(*L*, *E*_4_)]
[*E*_1_(*E*_4_(*)]	=	[*E*_1_(*E*_4_)] + [*R*(0, *E*_4_)] + [*R*(*L*, *E*_4_)]
		
[*R*(*L*, *E*_3_(*))]	=	[*R*(*L*, *E*_3_)] + [*R*(*L*, *E*_4_)]
[*R*(*, *E*_4_(*))]	=	[*R*(0, *E*_4_)] + [*R*(*L*, *E*_4_)]
		
[*R*(*L*, *E*_4_(*))]	=	[*R*(*L*, *E*_4_)]

The new states correspond to the occurrence levels of different subcomplexes. The transformation can be structured in different tiers. The previously discussed case of single protein ligand systems can be considered as border case of the underlying transformation pattern. The transformation is independent of the chosen output variables as well as the kinetic properties of the reaction network. However, another choice of output variables may lead to a higher or lower number of observable states. The same holds true for varying kinetic parameters. For given input and output signals the kinetic properties determine whether states are observable and/or controllable. Furthermore, the kinetic parameters also define whether the model equations can be modularized or not. In the considered example the system does not comprise unobservable states and can be divided into five modules if *k*_8 _≠ *k*_9 _and *k*_-8 _≠ *k*_-9_. If *k*_8 _= *k*_9 _and *k*_-8 _= *k*_-9 _the system can be reduced to ten ODEs.

Due to the absence of protein production and degradation the six states of the 0^th ^tier remain all constant. Thus, these six ODEs can be eliminated in the considered example. First, we will discuss the case that *E*_4 _binding is unidirectionally influenced by *E*_3 _binding. In this case, our transformation does not allow any exact reduction of the model, neither for the output variables ${\vec{y}}_{1}$ nor for ${\vec{y}}_{2}$. Interestingly, the transformed model equations can be dissected into five modules, which are all unidirectionally coupled. This model structure directly resembles the interaction pattern between the five considered binding processes. In fact each of the modules describes one of these five processes. However, the modules differ in size and structure.

The first module which describes the recruitment of *L *to the receptor only consists of one differential equation. The second, third, fourth and fifth module comprises two, three, four and five states, respectively. Another nice property of the transformed system is the concurrently achieved modularization of the kinetic parameters. For instance, the *L *binding module only contains the parameters *k*_1 _and *k*_-1_. In addition to *k*_1 _and *k*_-1_, the second module comprises all parameters that describe binding of *E*_1 _to *R *but no others. This special hierarchical structure is very advantageous for parameter estimation.

Measurements of the transient behavior either of the states ${\vec{y}}_{1}$ or ${\vec{y}}_{2}$ facilitate a stepwise identification of the kinetic model parameters module by module.

Taking the assumption that the association of *E*_3 _and *E*_4 _is independent of all other occurring binding processes the structure of the fifth module changes. The state *E*_3_(*E*_4_(*)) is not controllable any more, since the respective binding process can neither be directly nor indirectly influenced by changes in the *L *concentration. If ${\vec{y}}_{1}$ is the output vector of the system the output variable *E*_3_(*E*_4_(*)) is determined by the steady state equation of the respective ODE. The remaining four states of the fifth module are not observable and can be simply omitted. Thus, the model can be exactly reduced to ten ODEs. The situation changes if one considers the output vector ${\vec{y}}_{2}$. The choice of different output variables does not affect controllability of a system. Thus, the state *E*_3_(*E*_4_(*)) is still uncontrollable and the respective ODE can be replaced by its steady state equation. However, all model states are observable in this case, and thus no further equation can be eliminated. An exactly reduced model in this case would comprise fourteen ODEs.

##### Example 2: Domain phosphorylation

As a second example we consider a similar receptor *R*. In contrast to the previously considered example the receptor's intracellular domain has to be phosphorylated in order to bind the effector protein *E*_1_. Phosphorylation is considered to be a necessary precondition for *E*_1 _binding, while bound *E*_1 _preserves the receptor domain from dephosphorylation due to steric reasons. This corresponds to a so-called *all-or-none *interaction as it has been introduced by Koschorreck *et al*. [3]. *E*_1 _also has to be phosphorylated in order to recruit *E*_2_, which prevents dephosphorylation of *E*_1_. We do not consider binding of further effector proteins. The reaction rules for this system are given in Table 3.

**Table 3:** Reaction rules describing the Example depicted in Figure 2B.

*R*(0, *)	+	*L *	⇋	*R*(*L*, *)	*k*_1_, *k*_-1_
*R*(0, 0)			⇋	*R*(0, *P*)	*k*_2_, *k*_-2_
*R*(*L*, 0)			⇋	*R*(*L*, *P*)	*k*_3_, *k*_-3_
*R*(*, *P*)	+	*E*_1_(*)	⇋	*R*(*, *E*_1_(*))	*k*_4_, *k*_-4_
*E*_1_(0, 0)			⇋	*E*_1_(0, *P*)	*k*_5_, *k*_-5_
*R*(*, *E*_1_)			⇋	*R*(*, *E*_1_(*P*))	*k*_6_, *k*_-6_
*E*_1_(*, *P*)	+	*E*_2_	⇋	*E*_1_(*, *E*_2_)	*k*_7_, *k*_-7_

The kinetic parameters are specified behind the rules.

Again the concentration [*L*] is considered as the input of the system. The states [*R*(*L*, *)], [*R*(*, *P*)], [*R*(*, *E*_1_)], [*R*(*, *E*_1_(*P*))] and [*R*(*, *E*_2_)] are chosen as ouput variables $\vec{y}$. Since the system also comprises six different processes the transformation pattern again consists of six different tiers that are depicted in Table 4. Due to the absence of protein production and degradation the three states of the 0^th ^tier remain all constant. Thus, these three ODEs can be eliminated.

**Table 4:** Hierarchical transformation for the example system depicted in Figure 2B.

[*R*(*, *)]	=	[*R*(0, 0)] + [*R*(0, *P*)] + [*R*(0, *E*_1_)] + [*R*(0, *E*_1_(*P*))] + [*R*(0, *E*_2_)] + [*R*(*L*, 0)] + [*R*(*L*, *P*)] + [*R*(*L*, *E*_1_)] + [*R*(*L*, *E*_1_(*P*))] + [*R*(*L*, *E*_2_)]
[*E*_1_(*)]	=	[*E*_1_(0)] + [*E*_1_(*P*)] + [*E*_1_(*E*_2_)] + [*R*(0, *E*_1_)] + [*R*(0, *E*_1_(*P*))] + [*R*(0, *E*_2_)] + [*R*(*L*, *E*_1_)] + [*R*(*L*, *E*_1_(*P*))] + [*R*(*L*, *E*_2_)]
[*E*_2_(*)]	=	[*E*_1_(*E*_2_)] + [*E*_2_(0)] + [*R*(0, *E*_2_)] + [*R*(*L*, *E*_2_)]
		
[*R*(*L*, *)]	=	[*R*(*L*, 0)] + [*R*(*L*, *P*)] + [*R*(*L*, *E*_1_)] + [*R*(*L*, *E*_1_(*P*))] + [*R*(*L*, *E*_2_)]
[*R*(*, *P*(*))]	=	[*R*(0, *P*)] + [*R*(0, *E*_1_)] + [*R*(0, *E*_1_(*P*))] + [*R*(0, *E*_2_)] + [*R*(*L*, *P*)] + [*R*(*L*, *E*_1_)] + [*R*(*L*, *E*_1_(*P*))] + [*R*(*L*, *E*_2_)]
[*E*_1_(*P *(*)]	=	[*E*_1_(*P*)] + [*E*_1_(*E*_2_)] + [*R*(0, *E*_1_(*P*))] + [*R*(0, *E*_2_)] + [*R*(*L*, *E*_1_(*P*))] + [*R*(*L*, *E*_2_)]
		
[*R*(*L*, *P*(*))]	=	[*R*(*L*, *P*)] + [*R*(*L*, *E*_1_)] + [*R*(*L*, *E*_1_(*P*))] + [*R*(*L*, *E*_2_)]
[*R*(*, *E*_1_(*))]	=	[*R*(0, *E*_1_)] + [*R*(0, *E*_1_(*P*))] + [*R*(0, *E*_2_)] + [*R*(*L*, *E*_1_)] + [*R*(*L*, *E*_1_(*P*))] + [*R*(*L*, *E*_2_)]
[*E*_1_(*E*_2_(*)]	=	[*E*_1_(*E*_2_)] + [*R*(0, *E*_2_)] + [*R*(*L*, *E*_2_)]
		
[*R*(*L*, *E*_1_(*))]	=	[*R*(*L*, *E*_1_)] + [*R*(*L*, *E*_1_(*P*))] + [*R*(*L*, *E*_2_)]
[*R*(*, *E*_1_(*P *(*)))]	=	[*R*(0, *E*_1_(*P*))] + [*R*(0, *E*_2_)] + [*R*(*L*, *E*_1_(*P*))] + [*R*(*L*, *E*_2_)]
		
[*R*(*L*, *E*_1_(*P *(*)))]	=	[*R*(*L*, *E*_1_(*P*))] + [*R*(*L*, *E*_2_)]
[*R*(*, *E*_2_(*))]	=	[*R*(0, *E*_2_)] + [*R*(*L*, *E*_2_)]
		
[*R*(*L*, *E*_2_(*))]	=	[*R*(*L*, *E*_2_)]

The new states correspond to the occurrence levels of different subcomplexes. The transformation can be structured in different tiers. The previously discussed case of single protein ligand systems can be considered as border case of the unterlying transformation pattern.

In this example, the transformed model equations can be dissected into three unidirectionally coupled modules including all five output variables, and one additional module comprising the two unobservable states [*R*(*L, E*_1_(*P*))] and [*R*(*L, E*_2_)]. All model states are controllable. Thus, this example shows that the existence of all-or-none interactions facilitate significant model reductions. Although the considered system comprises the same number of molecular processes than the previously regarded one, even the complete mechanistic model already consists of a lower number of ODEs. Additionally, the system comprises two unobservable states, which allows for a further model reduction.

#### Homodimerization of receptors and scaffolds

Homodimerization of receptors and scaffold proteins is quite common in signal transduction networks. For instance, homodimers occur in the ErbB signaling network as described by Citri *et al*. [31].

Homodimerization is additionally characterized by a number of unique features having a strong impact on model reduction which justifies a separate and detailed discussion. Due to their symmetric configuration the number of distinguishable homodimers is much lower than of equally large heterodimers. If one considers a receptor monomer which forms *n *distinct monomeric multiprotein complexes there exist $\frac{n(n+1)}{2}$ feasible homodimers. Heterodimerization of two different receptors, which both form *n *monomeric species, leads to *n*^2 ^feasible heterodimers. However, the indistinguishability of symmetric receptor dimers not only has the positive effect of reducing the number of ODEs compared to heterodimers. It also leads to non-intuitive kinetic system properties, which will be discussed below.

We will consider a receptor *R *with *n *distinct binding domains. Furthermore, we presume that *R *can form homodimers. These homodimers shall be decipted as *R*(*, ..., *).*R*(*, ..., *). Due to the symmetry of the dimers one cannot distinguish between *R*(*L*, 0, ..., 0).*R*(0, ..., 0) and *R*(0, ..., 0).*R*(*L*, 0, ..., 0). Hence, we reach an agreement that the receptor with more occupied domains will always be noted first.

#### Kinetic properties

Dimerization is a molecular process such as ligand binding, and dimerization can influence or can be influenced by all other processes within the considered network. The most simple theoretic case one can analyze is that receptor homodimerization is completely independent of all other processes. In order to achieve this independence one has to parametrize all dimerization reactions adequately. This requires to distinguish between the formation of mirror symmetric dimers and non-mirror symmetric dimers. The reason for this discrimination is that reactions describing the formation of non-mirror symmetric dimers have to be parametrized by a twofold higher *k*_on _value than those of mirror symmetric dimers.

(11) $$\begin{array}{c} R(X_{1},...,X_{n})+R(X_{1},...,X_{n})\\ \overset{k_{1}}{\underset{k_{-1}}{⇋}}R(X_{1},...,X_{n}).R(X_{1},...,X_{n})\\ R(X_{1},...,X_{n})+R(Y_{1},...,Y_{n})\\ \overset{2k_{1}}{\underset{k_{-1}}{⇋}}R(X_{1},...,X_{n}).R(Y_{1},...,Y_{n}) \end{array}$$

The reason for this duplication of the *k*_on _value can be elucidated considering the reaction rates. Let us take the assumption that the two concentrations [*R*(*X*_1_, ..., *X*_*n*_)] and [*R*(*Y*_1_, ..., *Y*_*n*_)] are equal. The rates for the considered two reactions comprise the terms [*R*(*X*_1, _..., *X*_*n*_)]^2 ^and [*R*(*X*_1_, ..., *X*_*n*_)]·[*R*(*Y*_1_, ..., *Y*_*n*_)], respectively. According to the collision theory for chemical reactions these terms are measures for the likelihood of a collision of two reactants in the system. Due to our assumption that the concentrations of both species are equal, the evaluation of both terms leads to exactly the same numerical result. However, the likelihood for the formation of a non-mirror symmetric dimer is twofold higher than that for mirror symmetric ones. This becomes apparent if one considers the collision probability for both cases. In the second case the number of molecules that may collide is twofold higher than in the first one.

Not only the dimerization process itself and therefore the dimerization reactions have to be treated differently. Due to the symmetry of homodimers one also has to be careful in parametrizing ligand binding and modification reactions. Let us again assume that binding of the ligand *L *is completely independent of all other processes. Note, that this is a completely theoretic assumption in order to illustrate the occurring problems regarding the most simple scenario. Furthermore, let *k*_1 _and *k*_-1 _be the kinetic parameters describing the association and dissociation of *L *with a receptor monomer

(12) $$R(0,*,...,*)+L\overset{k_{1}}{\underset{k_{-1}}{⇋}}R(L,*,...,*).$$

Here, one has again to distinguish two cases, namely binding of *L *to a completely unliganded dimer and binding to a single liganded one. According to our assumption dimerization shall not have any effect on ligand binding. Hence, each receptor molecule of a dimer behaves exactly the same way as a monomeric receptor does, which indicates that an unliganded dimer has a twofold higher *k*_on _value than a single liganded or a monomeric one. The same rationale also implies that the *k*_off _value for a double liganded dimer is twofold higher than for a single liganded one. Thus, the reactions have to be parametrized as follows

(13) $$\begin{array}{l} L+R(0,*,...,*).R(0,*,...,*)\\ \overset{2k_{1}}{\underset{k_{-1}}{⇋}}R(L,*,...,*).R(0,*,...,*) \end{array}$$

(14) $$\begin{array}{l} L+R(L,*,...,*).R(0,*,...,*)\\ \overset{k_{1}}{\underset{2k_{-1}}{⇋}}R(L,*,...,*).R(L,*,...,*). \end{array}$$

The realization of process interactions either between two binding or modification processes or between dimerization and some other processes is straight forward. If dimerization has an influence on *L *binding, Reaction 12 will be parametrized by *k*_1 _and *k*_-1_, while the parameters *k*_2 _and *k*_-2 _will be used for the Reactions 13 and 14. However, one still has to account for the twofold higher association constant of Reaction 13 and the twofold higher dissociation constant of Reaction 14. The neglect of these additional factors corresponds to a mutual interaction between the two ligand binding processes within a dimer.

##### General transformation pattern

The general transformation for systems that include homodimerization follows exactly the same pattern as introduced for scaffolds with multiprotein ligands. It is hierarchically structured whereas the different tiers of the transformation comprise occurrence levels of one, two, three or higher molecule complexes. However, one has to be careful, since some of the species concentrations have to be counted twice. Let us consider the occurrence level of a receptor ligand complex, which we will depict as [*R*(*L*, *, ..., *).*]. This accumulated quantity comprises monomeric as well as dimeric species, namely [*R*(*L*, *, ..., *)], [*R*(*L*, *, ..., *).*R*(0, *, ..., *)] and [*R*(*L*, *, ..., *).*R*(*L*, *, ..., *)]. Observe, that the species *R*(*L*, *, ..., *).*R*(*L*, *, ..., *) include two receptor ligand complexes and therefore has to be counted twice. Consequently, the considered occurrence level is defined as

(15) $$\begin{array}{lll} \left[R(L,*,...,*).*\right] & = & \left[R(L,*,...,*)\right]\\ & & +[R(L,*,...,*).R(0,*,...,*)]\\ & & +2[R(L,*,...,*).R(L,*,...,*)]. \end{array}$$

The invertibility of the transformation matrix suggested here can again be proved using mathematical induction.

#### Example

As an example we will analyze homodimerization of the EGFR receptor which will be called *R *in the following. In addition to the dimerization process we also consider EGF binding and receptor phosphorylation. EGF binding and receptor dimerization are assumed to interact mutually. This assumption is in accordance with thermodynamic constraints [33,28], and also fits to experimental data presented by Odaka *et al*. and Lemmon *et al*. [34,35]. Furthermore, we assume that dimerization influences receptor phosphorylation, since the receptors of a dimer phosphorylate each other mutually. In analogy to the experimental results of Gherzi *et al*. [36] for insulin signaling this interaction is expected to be an unidirectional one. The reaction rules which describe this system are given in Table 5.

**Table 5:** Reaction rules for the considered example of EGFR dimerization.

*R*(0, *)	+	*EGF *	⇋	*R*(*EGF*, *)	*k*_1_, *k*_-1_
*R*(0, *).*R*(0, *)	+	*EGF *	⇋	*R*(*EGF*, *).*R*(0, *)	2*k*_2_, *k*_-2_
*R*(*EGF*, *).*R*(0, *)	+	*EGF *	⇋	*R*(*EGF*, *).*R*(*EGF*, *)	*k*_2_, 2*k*_-2_
*R*(0, *X*_1_)	+	*R*(0, *X*_1_)	⇋	*R*(0, *X*_1_).*R*(0, *X*_1_)	*k*_3_, *k*_-3_
*R*(0, *X*_1_)	+	*R*(0, *X*_2_)	⇋	*R*(0, *X*_1_).*R*(0, *X*_2_)	2*k*_3_, *k*_-3_
*R*(*EGF*, *X*_1_)	+	*R*(0, *X*_1_)	⇋	*R*(*EGF*, *X*_1_).*R*(0, *X*_1_)	*k*_4_, *k*_-4_
*R*(*EGF*, *X*_1_)	+	*R*(0, *X*_2_)	⇋	*R*(*EGF*, *X*_1_).*R*(0, *X*_2_)	2*k*_4_, *k*_-4_
*R*(*EGF*, *X*_1_)	+	*R*(*EGF*, *X*_1_)	⇋	*R*(*EGF*, *X*_1_).*R*(*EGF*, *X*_1_)	*k*_5_, *k*_-5_
*R*(*EGF*, *X*_1_)	+	*R*(*EGF*, *X*_2_)	⇋	*R*(*EGF*, *X*_1_).*R*(*EGF*, *X*_2_)	2*k*_5_, *k*_-5_
*R*(*, 0)			⇋	*R*(*, *P*)	*k*_6_, *k*_-6_
*R*(*, 0).*R*(*, 0)			⇋	*R*(*, *P*).*R*(*, 0)	2*k*_7_, *k*_-7_
*R*(*, *P*).*R*(*, 0)			⇋	*R*(*, *P*).*R*(*, *P*)	*k*_7_, 2*k*_-7_

Herein the identifiers *X*_*n *_also indicate that the related domains can be in various states as the identifier * does. However, all domains with the identifier *X*_*n *_within one rule have to be in the same state. If two different identifiers *X*_*i *_and *X*_*j *_occur within one rule the respective domains are not allowed to be in the same state.

The reaction system comprises 14 receptor species and the ligand EGF. The transformation of these states according to the proposed general transformation pattern is shown in Table 6. Since the concentration of extracellular EGF is considered as model input the transformation does not include the overall concentration of EGF. The most suggestive choice of output variables in this example are the three occurrence levels of the 1^st ^transformation tier, namely [*R*(*EGF*, *).*], [*R*(*, *P*).*] and [*R*(*, *).*R*(*, *)]. These outputs correspond to the total number of liganded EGF binding domains, the total number of phosphorylated intracellular receptor domains as well as the number of receptor dimers.

**Table 6:** Hierarchical transformation for the example system.

[*R*(*, *).*]	=	[*R*(0, 0)] + [*R*(*EGF*, 0)] + [*R*(0, *P*)] + [*R*(*EGF*, *P*)] + 2 [*R*(0, 0).*R*(0, 0)]
		+ 2 [*R*(*EGF*, 0).*R*(0, 0)] + 2 [*R*(0, *P*).*R*(0, 0)] + 2 [*R*(*EGF*, 0).*R*(*EGF*, 0)]
		+ 2 [*R*(*EGF*, *P*).*R*(0, 0)] + 2 [*R*(*EGF*, 0).*R*(0, *P*)] + 2 [*R*(0, *P*).*R*(0, *P*)]
		+ 2 [*R*(*EGF*, *P*).*R*(*EGF*, 0)] + 2 [*R*(*EGF*, *P*).*R*(0, *P*)] + 2 [*R*(*EGF*, *P*).*R*(*EGF*, *P*)]
		
[*R*(*EGF*, *).*]	=	[*R*(*EGF*, 0)] + [*R*(*EGF*, *P*)] + [*R*(*EGF*, 0).*R*(0, 0)] + 2 [*R*(*EGF*, 0).*R*(*EGF*, 0)]
		+ [*R*(*EGF*, *P*).*R*(0, 0)] + [*R*(*EGF*, 0).*R*(0, *P*)] + 2 [*R*(*EGF*, *P*).*R*(*EGF*, 0)]
		+ [*R*(*EGF*, *P*).*R*(0, *P*)] + 2 [*R*(*EGF*, *P*).*R*(*EGF*, *P*)]
[*R*(*, *).*R*(*, *)]	=	[*R*(0, 0).*R*(0, 0)] + [*R*(*EGF*, 0).*R*(0, 0)] + [*R*(0, *P*).*R*(0, 0)] + [*R*(*EGF*, 0).*R*(*EGF*, 0)]
		+ [*R*(*EGF*, *P*).*R*(0, 0)] + [*R*(*EGF*, 0).*R*(0, *P*)] + [*R*(0, *P*).*R*(0, *P*)]
		+ [*R*(*EGF*, *P*).*R*(*EGF*, 0)] + [*R*(*EGF*, *P*).*R*(0, *P*)] + [*R*(*EGF*, *P*).*R*(*EGF*, *P*)]
[*R*(*, *P*).*]	=	[*R*(0, *P*)] + [*R*(*EGF*, *P*)] + [*R*(0, *P*).*R*(0, 0)] + [*R*(*EGF*, *P*).*R*(0, 0)] + [*R*(*EGF*, 0).*R*(0, *P*)]
		+ 2 [*R*(0, *P*).*R*(0, *P*)] + [*R*(*EGF*, *P*).*R*(*EGF*, 0)] + 2 [*R*(*EGF*, *P*).*R*(0, *P*)]
		+ 2 [*R*(*EGF*, *P*).*R*(*EGF*, *P*)]
		
[*R*(*EGF*, *P*).*]	=	[*R*(*EGF*, *P*)] + [*R*(*EGF*, *P*).*R*(0, 0)] + [*R*(*EGF*, *P*).*R*(*EGF*, 0)] + [*R*(*EGF*, *P*).*R*(0, *P*)]
		+ 2 [*R*(*EGF*, *P*).*R*(*EGF*, *P*)]
[*R*(*EGF*, *).*R*(*, *)]	=	[*R*(*EGF*, 0).*R*(0, 0)] + 2 [*R*(*EGF*, 0).*R*(*EGF*, 0)] + [*R*(*EGF*, *P*).*R*(0, 0)]
		+ [*R*(*EGF*, 0).*R*(0, *P*)] + 2 [*R*(*EGF*, *P*).*R*(*EGF*, 0)] + [*R*(*EGF*, *P*).*R*(0, *P*)]
		+ 2 [*R*(*EGF*, *P*).*R*(*EGF*, *P*)]
[*R*(*, *P*).*R*(*.*)]	=	[*R*(0, *P*).*R*(0, 0)] + [*R*(*EGF*, *P*).*R*(0, 0)] + [*R*(*EGF*, 0).*R*(0, *P*)] + 2 [*R*(0, *P*).*R*(0, *P*)]
		+ [*R*(*EGF*, *P*).*R*(*EGF*, 0)] + 2 [*R*(*EGF*, *P*).*R*(0, *P*)] + 2 [*R*(*EGF*, *P*).*R*(*EGF*, *P*)]
		
[*R*(*EGF*, *).*R*(*EGF*, *)]	=	[*R*(*EGF*, 0).*R*(*EGF*, 0)] + [*R*(*EGF*, *P*).*R*(*EGF*, 0)] + [*R*(*EGF*, *P*).*R*(*EGF*, *P*)]
[*R*(*, *P*).*R*(*, *P*)]	=	[*R*(0, *P*).*R*(0, *P*)] + [*R*(*EGF*, *P*).*R*(0, *P*)] + [*R*(*EGF*, *P*).*R*(*EGF*, *P*)]
[*R*(*EGF*, *P*).*R*(*, *)]	=	[*R*(*EGF*, *P*).*R*(0, 0)] + [*R*(*EGF*, *P*).*R*(*EGF*, 0)] + [*R*(*EGF*, *P*).*R*(0, *P*)]
		+ 2 [*R*(*EGF*, *P*).*R*(*EGF*, *P*)]
[*R*(*EGF*, *).*R*(*, *P*)]	=	[*R*(*EGF*, 0).*R*(0, *P*)] + [*R*(*EGF*, *P*).*R*(*EGF*, 0)] + [*R*(*EGF*, *P*).*R*(0, *P*)]
		+ 2 [*R*(*EGF*, *P*).*R*(*EGF*, *P*)]
		
[*R*(*EGF*, *P*).*R*(*EGF*. *)]	=	[*R*(*EGF*, *P*).*R*(*EGF*, 0)] + 2 [*R*(*EGF*, *P*).*R*(*EGF*, *P*)]
[*R*(*EGF*, *P*).*R*(*, *P*)]	=	[*R*(*EGF*, *P*).*R*(0, *P*)] + 2 [*R*(*EGF*, *P*).*R*(*EGF*, *P*)]
		
[*R*(*EGF*, *P*).*R*(*EGF*, *P*)]	=	[*R*(*EGF*, *P*).*R*(*EGF*, *P*)]

The new states also correspond to the occurrence levels of different subcomplexes. Due to the symmetric structure of the recptor dimers some species have to be counted twice. For instance the macroscopic state [*R*(*EGF*, *).*] is an aggregation of all species that comprise a subcomplex consisting of one receptor and one EGF molecule. The two micro-states [*R*(*EGF*, 0).*R*(0, 0)] and [*R*(*EGF*, 0).*R*(*EGF*, 0)] obviously fit into this pattern. However, the state [*R*(*EGF*, 0).*R*(*EGF*, 0)] has to be counted twice since the regarded subcomplex also occurs twice in this species. Furthermore, the transformation can be structured in six different tiers.

Due to the absence of production and degradation, the overall concentration of EGFR stays constant and the respective ODE can be eliminated. The remaining 13 transformed model equations can be dissected into three modules. The first module consists of four ODEs and describes EGF binding as well as receptor homodimerization. It comprises the model states [*R*(*EGF*, *).*], [*R*(*, *).*R*(*, *)], [*R*(*EGF*, *).*R*(*, *)] and [*R*(*EGF*, *).*R*(*EGF*, *)]. The second module describes receptor phosphorylation and contains six ODEs, while the remaining three ODEs for [R(*, P).R(*.P)], [R(EGF, P).R(*, P)] and [R(EGF, P).R(EGF, P)] form the third unobservable module. Since all states are controllable the model can be reduced by omitting the three unobservable states. This reduced model then comprises ten ODEs.

#### Generality of the method

Nearly more important than the introduced method and the algorithm to exactly reduce a model is the question about its limitations. In this context, one has to distinguish between the limitations of exact reducibility and of the described reduction method. As we mentioned earlier the model structure as well as its input and output variables are mostly not discretionary. If a considered system is a minimal realization due to these characteristics it is impossible to find or develop any method which allows to exactly reduce this model. From our point of view this is no limitation of the method but of exact reducibility in general. It is difficult to make a descriptive but general statement under which conditions a combinatorial reaction network model is exactly reducible or not. However, one can state that retroactive effects as well as feedbacks can strongly reduce the number of eliminable states. If at least one of the binding or modifications processes that are of interest is directly or indirectly affected by all other considered processes the chances to reduce such a system exactly are very low. From a mathematical point of view one can make much more general statements. Control theory provides numerous techniques which allow to check any system for observability or controllability. If all model states are observable and controllable the application of our method is not convenient instead one should alternatively use an approximative reduction technique like proposed by Koschorreck *et al*. [3]. One possibility is to check for local observability or controllability of the reduced models by analyzing the linearized model equations

(16) $$\begin{array}{l} \dot{\vec{x}}=A\vec{x}+B\vec{u}\\ \vec{y}=C\vec{x} \end{array}$$

with $A={\left(\frac{\partial \vec{f}(\vec{x},\vec{u})}{\partial \vec{x}}\right)}_{{\vec{x}}_{o},{\vec{u}}_{o}}$, $B={\left(\frac{\partial \vec{f}(\vec{x},\vec{u})}{\partial \vec{u}}\right)}_{{\vec{x}}_{o},{\vec{u}}_{o}}$ and $\vec{x}$ ∈ ℝ^*n*^, $\vec{u}$ ∈ ℝ^*m*^. If all states of the linearized model are controllable and observable this proves that at least at the considered operating point (${\vec{x}}_{o}$, ${\vec{u}}_{o}$) all states are required to accurately describe the systems behavior and that the model is not exactly reducible. The system is said to be *locally controllable *at the operating point if the rank of the matrix P (see Equation 9) is *n *[25]. Accordingly, the system is said to be *locally observable *if the rank of the matrix Q (see Eqnation 7) is *n *[25]. However, using this analysis method one has to be aware of the fact that controllable and observable nonlinear system states might loose controllability and/or observability at individual operating points [25]. A matrix rank of *n *proves that the considered system cannot be exactly reduced. At least at the chosen operating point all states are controllable and therefore affect the system's input/output behavior. If a model still comprises uncontrollable or unobservable states, the rank of the related matrix will be smaller than *n *for all considered operating points. However, if the matrix rank is lower than *n *this is no proof that the system is exactly reducible and still comprises uncontrollable or unobservable states. It is also possible that the system was linearized at an unpropitiously chosen operating point. Hence, it might be necessary to repeat the test at several operating points. A matrix rank smaller than *n *for numerous operating points highly suggests the further existence of uncontrollable or unobservable states in the nonlinear system. However, it is no proof.

After having discussed the limitations of exact reducibility in general, we also want to address the limitations of our method. A limitation of an exact reduction method is it does not facilitate the reduction of an exactly reducible model. Unfortunately, it is not possible to generally prove that our method has or has not such limitations. However, each model that has been reduced using our method can be linearized and checked for local observability and controllability. If the rank of the corresponding *P *and *Q *matrices is *n *all unobservable and uncontrollable system states have been eliminated.

All examples discussed in Conzelmann *et al*. [16] as well as the examples discussed above have been checked for further uncontrollable or unobservable states using this approach. In all cases, the reduced models proved to be minimal realizations even for varying input and output variables. However, there exists an interesting border case, in which a model can be further reduced without affecting the input/output behavior. This border case shall be discussed below.

We consider a receptor with three binding domains whereas one extracellular domain controls the two intracellular domains in an unidirectional manner. This example is also discussed by Conzelmann *et al*. [16]. However, in contrast to the example discussed there, we presume that the two intracellular domains are identical. Both recruit the same effector protein *E *and both have exactly the same kinetic properties. Let us further assume that the system output is the total number of *E *proteins bound to the receptor, which corresponds to the sum of both occupancy levels. The proposed transformation facilitates the elimination of the two unobservable ODEs [*R*(*, 1, 1)] and [*R*(1, 1, 1)] (see also Conzelmann *et al*. [16]). In this case, the remaining ODEs can only be dissected into two modules. Although the two identical domains do not interact with each other their ODEs are coupled due to the fact that both recruit the same effector. The module that describes the two intracellular domains resembles the symmetry of the considered system. Its equations form two identical but coupled submodules

(17) $$\begin{array}{cc} {\dot{\vec{x}}}_{1}=\vec{f}({\vec{x}}_{1},{\vec{x}}_{2},\vec{u}) & {\vec{x}}_{1}(0)={\vec{x}}_{0,1},\\ {\dot{\vec{x}}}_{2}=\vec{f}({\vec{x}}_{2},{\vec{x}}_{1},\vec{u}) & {\vec{x}}_{2}(0)={\vec{x}}_{0,2},\\ y=C({\vec{x}}_{1}+{\vec{x}}_{2}), & \end{array}$$

each describing one of the two identical binding domains. However, note that the initial conditions do not necessarily have to coincide. Under these assumptions the system still comprises unobservable states if the vector field $\vec{f}$ fulfills the superposition principle

(18) $$\begin{array}{l} \vec{f}({\vec{x}}_{1},{\vec{x}}_{2},\vec{u})+\vec{f}({\vec{x}}_{2},{\vec{x}}_{1},\vec{u})=\\ \vec{f}({\vec{x}}_{1}+{\vec{x}}_{2},{\vec{x}}_{1}+{\vec{x}}_{2},\vec{u})=\vec{g}({\vec{x}}_{1}+{\vec{x}}_{2},\vec{u}). \end{array}$$

In this case the system output *y *and its derivatives only depend on the sum of ${\vec{x}}_{1}$ and ${\vec{x}}_{2}$

(19) $$\dot{y}=C\left(\vec{f}({\vec{x}}_{1},\vec{u})+\vec{f}({\vec{x}}_{2},\vec{u})\right)=C\vec{g}({\vec{x}}_{1}+{\vec{x}}_{2},\vec{u}).$$

Thus, a minimal realization of the system would be

(20) $$\begin{array}{l} \begin{array}{ll} \dot{\vec{\xi }}=\vec{g}(\vec{\xi },\vec{u}) & \vec{\xi }(0)={\vec{\xi }}_{0}={\vec{x}}_{10}={\vec{x}}_{20} \end{array}\\ y=C\vec{\xi }. \end{array}$$

The superposition principle is fulfilled if the operator $\vec{f}$ is linear in $\vec{x}$. In the more general case if $\vec{f}$ does not fulfill the superposition principle our transformation provides a minimal realization of the system. However, note that even for a general operator $\vec{f}$ the number of equations can be reduced if the initial conditions of both submodules are equivalent $\left({\vec{x}}_{10}={\vec{x}}_{20}\right)$. Under this condition both submodules are completely identical $\left({\vec{x}}_{1}={\vec{x}}_{2}=\vec{\xi }\right)$ and therefore one of them can be eliminated and the reduced module can be written as

(21) $$\begin{array}{l} \begin{array}{ll} \dot{\vec{\xi }}=\vec{f}(\vec{\xi },\vec{\xi },\vec{u})=\vec{g}(\vec{\xi },\vec{u}) & \vec{\xi }(0)={\vec{\xi }}_{0}={\vec{x}}_{10}={\vec{x}}_{20} \end{array}\\ y=2C\vec{\xi }. \end{array}$$

However, this reduction is not due to the elimination of unobservable states as defined above but results from the restricted choice of initial conditions. From these considerations it can be seen that except for the case of two identical linear subsystems no example has been found for which the proposed transformation does not provide a Kalman decomposition.

### Reduced order modeling of combinatorial reaction networks

In the previous section, we discussed a general and systematic method that allows for significant and exact model reductions of combinatorial reaction networks. Now, an alternative approach shall be considered that facilitates the direct generation of the exactly reduced model equations. This reduced order modeling approach is based on the close relations between controllability and observability of a model and the process interactions of the examined system.

#### Controllability, observability and process interactions

From the previously regarded examples it can be seen that the number of observable and controllable states highly depends on the occurring process interactions. The question is whether the qualitative information about process interactions can give clues about the observability and controllability of a reaction network, or maybe even facilitate a direct translation to reduced model equations. Controllability and observability as well as process interactions provide information about interactions within the considered system, however, at different levels of abstraction.

Controllability and observability are properties of an ODE system, and both of them characterize the ODE couplings with respect to the system inputs and outputs. All observable states exert a certain influence on at least one of the output variables. On the other hand a state is said to be controllable, if it can be influenced either directly or indirectly by one of the system's input variables.

Process interactions describe the regarded system at a different level of abstraction. However, they also provide information about which processes can influence other processes and which can be influenced by other processes. Controllability and observability are closely related to the definition of input and output variables, respectively. In accordance to this definition at the ODE level, one can also formally define input and output processes at the process level. A connection between the two abstraction levels is given by the occurrence levels we previously introduced as a state space representation for combinatorial reaction networks. These coordinates allow a direct assignment of model states to specific molecular processes.

Each occurrence level like e.g. [*R*(*, ..., *, *E*_*i*_, *..., *)] can be directly assigned to its respective process, namely *E*_*i *_binding to *R*. Analogously, occurrence levels of higher tiers like [*R*(*E*_1_, *E*_2_, *E*_3_, *, ..., *)] can be linked with three different processes. All processes that are related to the chosen output variables are said to be output processes, and all processes that can be directly assigned to the input variables analogously correspond to the input processes. This direct link between model variables and processes facilitates the unique translation of all input and output variables to a set of input and output processes. Let us consider an example and presume that the concentration of *E*_1 _is regarded as input variable while [*R*(*, *E*_2_, *, ..., *)] and [*R*(*, *, *, *E*_4_, *E*_5_, *, ..., *)] are output variables. In this case *E*_1 _binding to *R *is an input process, and *E*_2_, *E*_4 _and *E*_5 _binding to *R *are output processes.

Furthermore, we can formally introduce *process controllability *and *process observability*. A process shall be called process controllable if it is either directly or indirectly influenced by one of the input processes. Analogously, a process will be called process observable if it directly or indirectly affects one of the output processes. In contrast to controllability and observability of an ODE model, the respective system properties at the process level can be analyzed in a very simple way by considering the process interaction graph. In this graph processes are regarded as nodes, while process interactions are represented as directed edges. This definition of an interaction graph is very similar to that proposed by Klamt *et al*. [37]. A process *P *is process controllable if the interaction graph comprises a directed path from one of the input processes to the process *P*. The same process is observable if there exists a directed path from *P *to one of the output processes.

A relation between the controllability and observability concepts at the different abstraction levels can also be approved. Process controllability suggests that all states that are assigned to this process are influenced and therefore controllable. Process observability on the other hand indicates that the respective occurrence level of the 1^st ^tier is observable. State variables that describe occurrence levels of higher tiers, as [*R*(*E*_1_, *E*_2_, *E*_3_, *, ..., *)], are only observable if the related processes all jointly affect at least one of the output processes. Thus, we have found a way to predict whether a certain state might be observable or controllable by considering the process interactions. Note, that this technique provides a conservative appraisal which in some cases will classify states as controllable or observable although they are not.

#### Reduced order modeling technique

**Figure 3:**
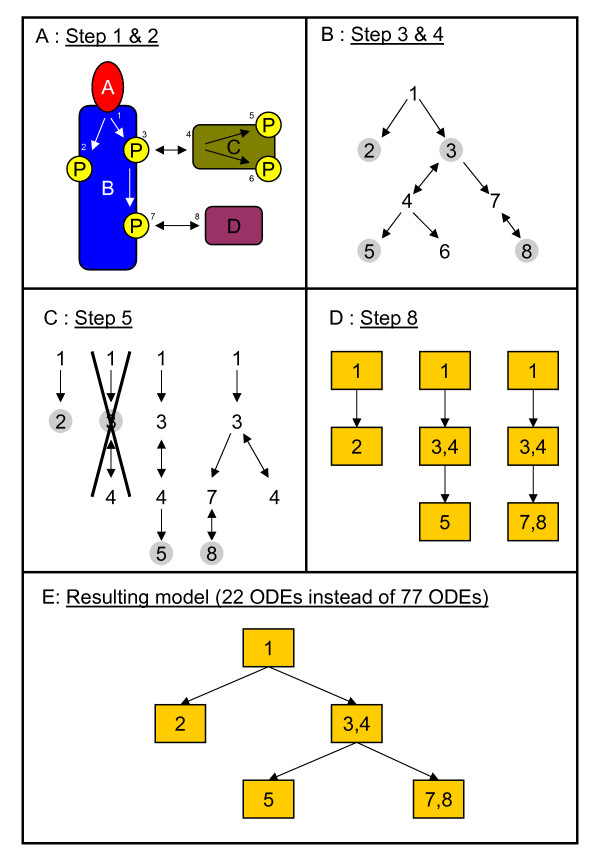
Exemplification of the developed reduced order modeling technique. The considered example is very similar to the previously discussed insulin example. Only the interaction pattern is a bit different. The depicted steps of the reduced order modeling technique are explained in the text.

The enormous complexity of most real signal transduction networks often impedes the application of common model reduction techniques discussed in literature as well as the previously proposed model reduction concept. New alternative techniques are required that allow the direct generation of reduced model equations. The already introduced concepts of process interactions, interaction graphs as well as process controllability and observability serve as a basis for the following considerations. The fundamental idea is that at the macroscopic level a mathematical description of a certain process merely requires the incorporation of those other processes that exert some influence on the considered one. A detailed specification of the method will be given in the following and is structured in nine elementary steps. Each step will be illustrated considering the example shown in Figure 3.

**Step 1: **Definition of all proteins, binding domains as well as binding and modification processes that shall be included to the model.

**Example: **In the considered example the model will comprise the molecules *A*, *B*, *C *and *D *with their binding domains as depicted in Figure 3A. The occurring processes are usually labelled or numbered like indicated in Figure 3A. In the example we consider eight different processes, namely binding of *A *to *B *(process 1), phosphorylation of *B *at different domains (processes 2, 3 and 7), binding of *C *to *B *(process 4), phosphorylation of *C *at two distinct domains (processes 5 and 6) as well as binding of *D *(process 8).

**Step 2: **In a second step one has to define all occurring process interactions and whether these are uni- or bidirectional. These process interactions have to be consistent with both measured kinetic data of the involved proteins and the thermodynamic constraints as discussed in Ederer *et al*. and Conzelmann *et al*. [38,28]. These constraints for instance highly restrict the possible occurrence of unidirectional interactions. Nature can only realize unidirectional interactions at the expense of energy [28]. Since a mathematical model requires a complete definition for all interactions, mostly fragmentary knowledge has to be completed by assumptions.

**Example: **In Figure 3A the occurring process interactions of the example are indicated by arrows. The processes (1, 2), (1, 3), (3, 7), (4, 5) and (4, 6) are assumed to interact unidirectionally. This assumption is in accordance with the mentioned thermodynamic constraints since phosphorylation consumes energy rich ATP [28]. The processes (3, 4) and (7, 8) are regarded as all-or-none interactions which by definition are mutual ones. All other processes do not interact directly.

**Step 3: **The interaction pattern of the system has to be translated into an interaction graph. As mentioned before the labelled or numbered processes are nodes and the occurring interactions are represented by directed edges (arrows) pointing to the process which is influenced.

**Example: **The interaction graph for the considered example is depicted in Figure 3B.

**Step 4: **One defines input and output processes according to the considered system stimulations as well as available measurements or research interests. If for instance the external ligand concentration is considered as input the related input process is ligand binding. If one is interested in receptor phosphorylation all phosphorylation processes at the receptor have to be chosen as output processes. The goal of generating a model that accurately describes the output processes at a macroscopic level necessitates the further inclusion of all other processes that are process observable.

**Example: **In the example we choose process 1 an input and the processes 2, 3, 5 and 8 as output processes. The output processes are marked by grey circles in Figure 3B.

**Step 5: **The interaction graph can be divided into output subgraphs. An output subgraph contains all nodes from which a specific output node can be reached following the directed edges. The most simple way of finding an output subgraph is to invert the directions of all arrows in the interaction graph and to start at one output process. Following the arrows one marks all processes that are directly connected with the output process. Afterwards one marks all other processes that are directly linked with the previously found processes. This procedure is repeated until one either reaches dead end processes or the number of marked processes does not increase anymore. The so found output subgraph comprises all processes, which are process observable considering the chosen output process. If a node does not occur in any output subgraph the corresponding process cannot influence any of the output processes and can be completely omitted in the following. Finally, one has to eliminate redundant information, i.e. subgraphs which are completely comprised in other bigger subgraphs. In principle one also can analogously define input subgraphs and determine which processes are uncontrollable. In this case one does not have to invert the directions of the interaction arrows. One simply uses the original directed graph, starts at an input process and performs exactly the same procedure as described for output processes. However, uncontrollable but observable processes cannot simply be eliminated from further consideration. Uncontrollability merely allows for steady state assumptions at the ODE level.

**Example: **The graph shown in Figure 3B can be divided into four output subgraphs as shown in Figure 3C. In this example process six does not influence any of the considered output processes and can be omitted in the following considerations. The subgraph for output process three is completely comprised in two other subgraphs and therefore can be eliminated. In this example there only exists one input subgraph that comprises all processes.

**Step 6: **Each of the output subgraphs describes an autonomous signaling path, which can be modeled separately. Note, that the first five steps are independent of the used modeling approach. For instance, one can also generate stochastic models on the basis of the derived output subgraphs. However, we will focus on ODE models now. Hence, the next step is to create complete mechanistic ODE models for each subgraph. Processes not being part of a subgraph are not included in the respective model.

**Example: **This step shall be exemplified considering the smallest subgraph of the example system that comprises the processes 1 and 2. The mathematical model is given by

(22) $$\begin{array}{l} r_{1}=k_{1}[A][B(0,0)]-k_{-1}[B(A,0)]\\ r_{2}=k_{1}[A][B(0,P)]-k_{-1}[B(A,P)]\\ r_{3}=k_{2}[B(0,0)]-k_{-2}[B(0,P)]\\ r_{4}=k_{3}[B(A,0)]-k_{-3}[B(A,P)] \end{array}$$

(23) $$\frac{d}{dt}\left(\begin{array}{c} \left[A\right]\\ \left[B(0,0)\right]\\ \left[B(A,0)\right]\\ \left[B(0,P)\right]\\ \left[B(A,P)\right] \end{array}\right)=\left(\begin{array}{c} -r_{1}-r_{2}\\ -r_{1}-r_{3}\\ r_{1}-r_{4}\\ -r_{2}+r_{3}\\ r_{2}+r_{4} \end{array}\right),$$

in which the rates *r*_1 _and *r*_2 _describe the binding of *A *to the scaffold protein B (process 1), and the rates *r*_3 _and *r*_4 _describe the phosphorylation of *B *(process 2).

**Step 7: **The model equations have to be transformed to new more convenient coordinates, which allow to eliminate redundant information still included in the subgraphs. This redundancy is due to the fact that some processes are comprised in several subgraphs. A suitable choice of new coordinates is given by the previously introduced occurrence levels. Note, that we highly recommend to choose always the transformation patterns discussed in the previous sections since these guarantee that the redundant information can be eliminated. However, we also want to state that there exist numerous other transformations that also would allow to eliminate the redundant information.

**Example: **As an example we again consider only the smallest subgraph of the system which comprises the processes 1 and 2. The first tier in this example includes the overall concentrations of the molecules *A *and *B*

(24) $$\begin{array}{c} \left[A(*)]=[A]+[B(A,0)]+[B(A,P)\right]\\ \left[B(*,*)]=[B(0,0)]+[B(A,0)]+[B(0,P)\right]\\ +[B(A,P)]. \end{array}$$

The next tier comprises the first order occurrence levels, which are given by

(25) $$\begin{array}{c} \left[B(A,*)]=[B(A,0)]+[B(A,P)\right]\\ {}[B(*,P)]=[B(0,P)]+[B(A,P)]. \end{array}$$

In this example there only occurs one further tier describing the second order occurrence levels, namely

(26) [*B *(*A, P*)] = [*B *(*A, P*)].

Processes that are not included in the currently considered subgraph are simply omitted since they are not observable. If the sub-model still contains unobservable states these can also be eliminated at this stage of the procedure.

**Step 8: **The proposed transformation allows to dissect the model equations of each subgraph into modules like shown above. These modules are characterized by unidirectional communication with other modules. Processes which directly or indirectly interact mutually form one module. If some processes are included in more than one subgraph, the models of these subgraphs will contain identical modules. Multiple copies of modules can be eliminated and the remaining modules can be merged to a complete model.

**Example: **For instance, the transformed ODEs for the discussed smallest subgraph do have a special structure. The variables [*A*(*)] and [*B*(*, *)] are constant and equal their initial concentration. The corresponding ODEs are not required. Additionally, the ODE for [*B*(*A*, *)] does not depend on [*B*(*, *P*)] and [*B*(*A, P*)], which is due to the unidirectional process interaction between *A *binding to *B *and phosphorylation of *B*. Hence, the remaining three ODEs can be divided into two modules. One module only comprises the ODE for [*B*(A, *)], which describes the dynamics of process 1. The second module comprises the other two ODEs, which describe the dynamics of process 2. The ODEs deduced from the two remaining output subgraphs shown in Figure 3C, can be divided into six more modules as indicated in Figure 3D. Each box represents a set of ODEs. The modules are labelled with the process numbers which are described by the appropriate ODEs. Two copies of module (1) and one of module (3,4) can be eliminated here. The resulting model, which consists of only 22 ODEs, is schematically shown in Figure 3. A complete mechanistic model of the exemplified network would comprise 77 ODEs of which three can be eliminated due to mass conservation relations.

**Step 9: **In a last step one can take a steady state assumption for all uncontrollable states that are still part of the reduced model. Note that this step is not obligatory and in some cases can be problematic since the steady state equations have to be solved. If it is possible it is advantageous to solve these equations analytically. However, in many cases an analytic solution might be unfeasible and the steady state equations have to be solved numerically in the step of numerical integration which then necessitates a DAE (Differential Algebraic Equation system) solver.

**Example: **In the regarded example all states are controllable and therefore no steady state assumptions can be made.

**Figure 4:**
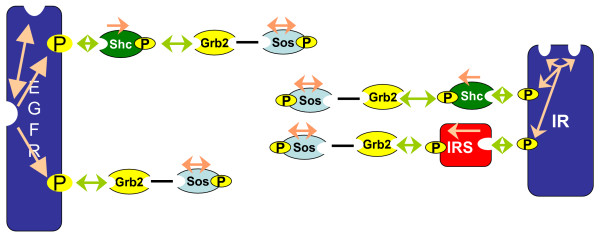
The shown part of the EGF and insulin receptor network is modeled. The process interactions are depicted by arrows. Black arrows represent uni- and bidirectional interactions, while grey arrows describe all-or-none interactions. A complete mechanistic model of this network comprises 5,182 ODEs, while the exactly reduced one consists of only 87 ODEs.

The main advantage of the proposed method is the direct generation of a reduced, but exact, system of equations, circumventing a unsuitable large model of full combinatorial complexity. Admittedly, the number of equations that has to be set up in step six mostly include redundant information about processes which can be observed at numerous outputs. However, the absolute number of ODEs that have to be generated is usually much lower than if a complete mechanistic model is created. In the considered example one only has to set up 27 ODEs using the described procedure. A complete combinatorial model would comprise 77 states. The method has to be slightly modified if one of the output variables is a higher order occurrence level which is not contained in any of the submodels. Let us assume that one of the output variables in the example is [*B*(*, *P*(*), *P*(*), *)] which describes both process 2 and process 3. Since none of the three subgraphs depicted in Figure 4C comprises both processes simultaneously the quantity [*B*(*, *P*(*), *P*(*), *)] will not be a state of the reduced 22 ODE model. This problem can be overcome by the fusion of two subgraphs. This will necessarily increase the number of ODEs that has to be generated as well as the final size of the reduced model. However, the number of ODEs would still be smaller than 77. Furthermore, the inclusion of production and degradation into the mathematical model necessitates another extension of this method. The same holds true if the regarded system includes multifunctional protein binding domains, i.e. that certain binding domains are involved in several binding processes. Both cases shall be discussed in the following. Note, that these problems do not occur if a the method described in Section *Exact Model Reduction *is used.

#### Multifunctional protein binding domains

Multifunctional protein binding domains are domains which can recruit more than one binding partner. A typical example is the effector protein Grb2 that can either bind to several ErbB receptors as well as to the adaptor protein Shc [39]. A constellation like this can lead to problems with the reduced order modeling approach introduced above. The problem occurs if such a multifunctional binding domain is part of two or more output subgraphs as shown in Figure 3C.

The probably most simple example to illustrate the occurring problem is a scaffold protein *R *which provides two binding domains. Both of these domains shall recruit the effector protein *E *which possesses one binding domain. The binding domain of *E *is a multifunctional one since it can bind to both *R *domains. If we assume that the two binding processes of the regarded system are completely independent and that both are considered as output processes, the system can be divided into two subgraphs. These subgraphs are somehow degenerated since both only comprise a single node. According to the reduced order modeling approach both binding processes can be modeled separately. However, the problem is that the binding domain of the effector *E *is involved in both processes. This is a typical crosstalk situation. Since the number of effector proteins *E *and therefore the number of *E *binding domains is limited, the recruitment of *E *to one receptor domain reduces the concentration of unbound effector and therefore has an indirect influence on the other binding process.

One possible solution for this problem is to merge all output subgraphs that share such multifunctional binding domains. This approach has the drawback that the number of equations that have to be generated in the sixth step of the modeling procedure can be significantly increased. Alternatively, one can formulate the reaction rates for both subgraphs independently. However, all species which are simultaneously involved in both submodels have to be balanced in one joint ODE. If the first subgraph of the regarded example is translated into a reaction rate one has to consider only the rate

(27) *r*_1 _= *k*_1 _[*R*(0, #)] [*E*] - *k*_-1 _[*R*(*E*, #)].

In this representation the identifier # indicates that the real scaffold protein offers further binding domains but that the resulting combinatorial complexity is neglected. The second subgraph can be described by the reaction rate

(28) *r*_2 _= *k*_2 _[*R*(#, 0)] [*E*] - *k*_-2 _[*R*(#, *E*)].

An ODE model is obtained by balancing all occurring species. Since the species *E *is involved in both submodels one has to create one joint ODE for [*E*]. Note that species like *R*(0, #) and *R*(#, 0) are considered to be completely different molecules. The resulting ODE model is given by

(29) $$\begin{array}{c} \begin{array}{cc} \frac{d[R(0,\#)]}{dt}=-r_{1} & \frac{d[R(E,\#)]}{dt}=r_{1} \end{array}\\ \begin{array}{cc} \frac{d[R(\#,0)]}{dt}=-r_{2} & \frac{d[R(\#,E)]}{dt}=r_{2} \end{array}\\ \frac{d[E]}{dt}=-r_{1}-r_{2}. \end{array}$$

Following this procedure one does not have to consider the complete combinatorial complexity of the network. One also has to use a joint transformation which in this case is given by

(30) $$\begin{array}{c} \left[R(*,\#)]=[R(0,\#)]+[R(E,\#)\right]\\ \left[R(\#,*)]=[R(\#,0)]+[R(\#,E)\right]\\ \left[E(*)]=[E]+[R(E,\#)]+[R(\#,E)\right]\\ \left[R(E,\#)]=[R(E,\#)\right]\\ {}[R(\#,E)]=[R(\#,E)]. \end{array}$$

This means that occurrence levels can be composed of species from both submodels like [*E*(*)] in the regarded example.

This simple modification or extension of the proposed modeling approach facilitates its application to a larger set of reaction systems. It will also be of great importance in modeling the crosstalk between EGF and insulin receptor discussed below.

#### Production and degradation

A process that has been completely neglected in the preceding considerations which, however, plays a crucial role in many real signal transduction networks is production and degradation of signaling proteins. This process increases or decreases the number of available proteins. A quite simple way of modeling production and degradation, which we will adopt here, is the assumption of a constant production rate and a degradation rate which is proportional to the concentration of the degraded species. In many cases ubiquitin is used as marker for controlled degradation as for example shown for ErbB1 receptors [31]. A still unanswered question in this context is whether the whole signaling complex is degraded or only the ErbB receptor while the associated adaptor proteins are recycled.

For the sake of simplicity we take a number of assumptions. First, the considerations shall be focused on production and degradation of the regarded receptor or scaffold protein and its complexes. The individual receptor protein *R *shall be produced with a constant rate and all receptor species are presumed to have a natural decay rate. All other adaptor and effector proteins are neither produced nor degraded. If a receptor complex is degraded all bound adaptor proteins shall be recycled to the cytosol. Furthermore, we take the assumption that if the receptor is marked by ubiquitination its degradation rate is modulated. This change of the degradation rate from natural decay to ordered degradation can be considered as a process interaction. Ubiquitination has a direct influence on degradation. It is quite obvious that all processes that involve one of the *R *binding domains are affected by the considered production and degradation.

Theoretically, degradation can be considered as a process which sets the *k*_on _values of all *R *binding domains to zero and all *k*_off _values to infinity. All other effects caused by degradation are indirect effects. Note that if one takes the assumption that a complex is degraded with all its bound adaptor proteins all processes that modify or enlarge the *R *complex are directly influenced. All these interactions are unidirectional ones, which can be simply introduced in the process interaction graph. Production and degradation is one additional node in this graph which is influenced by ubiquitination and affects numerous other processes.

### Example: EGF and insulin receptor crosstalk

Finally, the discussed methods shall be used to generate a reduced model of EGF and insulin receptor crosstalk. We will compare a complete mechanistic description of this crosstalk and an exactly reduced version.

#### Model definition

In a first step it shall be defined which molecules and processes are included to the model and what assumptions are made concerning the process interactions. Since a complete mechanistic model that is still manageable shall also be generated the considerations will be limited to a small part of the real signaling network. For instance only the EGF receptor (EGFR) will be taken into account and the other three ErbB receptors shall be neglected. Similar simplifications were made by many other modelers in the past years [12,10,27]. In order to avoid an unmanageable combinatorial explosion of feasible EGF receptor species only two intracellular domains will be modeled. According to Schulze *et al*. the EGF receptor possesses among others six potential residues for Grb2 and also six residues for Shc binding [40]. Hence, we consider one binding domain for each of these two effector proteins. Concerning the insulin receptor family we will focus on the insulin receptor (IR) and exclude potential crosstalk with the insulin-like growth factor receptor (IGFR) and the insulin related receptor (IRR) [41]. Again we restrict the considerations to two intracellular IR domains, namely one for Shc and one for IRS [30].

EGFR provides an extracellular binding domain that recruits EGF [31,42]. Furthermore, the receptor monomers can form homodimers after being activated by the ligand. This dimerization induces phosphorylation of numerous intracellular domains [43-45]. According to thermodynamic constraints [33,28], EGF binding and receptor dimerization have to interact mutually fulfilling the Wegscheider conditions. A mutual interaction is also suggested by experimental data [46,47].

Phosphorylation can be unidirectionally influenced like discussed for the insulin receptor by Gherzi *et al*. [36]. Analogously, EGFR dimerization is assumed to unidirectionally influence EGFR autophosphorylation of the regarded intracellular domains. A direct interaction between EGF binding and phosphorylation is not presumed to occur. After the two intracellular domains are phosphorylated one of them recruits Grb2 and the other Shc [40]. The interaction between receptor phosphorylation and subsequent effector binding shall be an all-or-none interaction. Furthermore, it is also known that Shc can be phosphorylated after having bound to EGFR [39]. Shc binding is thought to unidirectionally affect Shc phosphorylation. The phosphorylated Shc protein can also recruit Grb2 [39]. Grb2 possesses an additional domain which recruits the adaptor protein SOS. SOS is a guanine exchange factor (GEF) which can activate the membrane bound small G-protein Ras by effecting the exchange of GDP for GTP [48,49].

Active RasGTP in turn initiates the MAP kinase cascade. Phosphorylated ERK which is a component of the MAP kinase cascade stimulates a serine/threonine phosphorylation of SOS resulting in dissociation of the Grb2-SOS complex [50,49]. Thus, we take the assumption that the Grb2-SOS binding is not influenced by Grb2 association to phosphorylated EGF receptor or phosphorylated Shc. However, if SOS is phosphorylated by ERK, which is considered as additional input signal, the Grb2-SOS complex dissociates. Here we assume a mutual interaction between SOS phosphorylation and Grb2-SOS binding.

The insulin receptor consists of two constitutively dimerized monomers and is activated exclusively by ligand binding without further oligomerization [30]. Due to the dimeric structure of the insulin receptor two insulin binding domains will be included to the model. According to the thermodynamic constraints and experimental results these two domains have to interact mutually [51]. Ligand binding is assumed to unidirectionally influence the phosphorylation of the two regarded intracellular domains [36,28]. Shc is assumed to bind with other kinetic parameters to IR than to EGFR. However, Shc phosphorylation, Grb2 binding to phosphorylated Shc etc. is parametrized like in the case of EGFR. In order to reduce the complexity of the network numerous binding domains of the scaffold IRS are neglected. The model only accounts for IRS binding to the phosphorylated insulin receptor, subsequent IRS phosphorylation and binding of the Grb2-SOS complex. In order to reduce the complexity of the model, receptor internalization and degradation is also neglected for both IR and EGFR.

All considered molecules, processes and process interactions are also depicted in Figure 4. The reaction rules generating this complete mechanistic model are depicted in Table 7.

**Table 7:** Reaction rules for the considered example of EGF and insulin receptor crosstalk.

*IR*(0, 0, *, *)	+	*Ins *	⇋	*IR*(*I*, 0, *, *)	*k*_1_, *k*_-1_
*IR*(0, 0, *, *)	+	*Ins *	⇋	*IR*(0, *I*, *, *)	*k*_1_, *k*_-1_
*IR*(*I*, 0, *, *)	+	*Ins *	⇋	*IR*(*I*, *I*, *, *)	*k*_2_, *k*_-2_
*IR*(0, *I*, *, *)	+	*Ins *	⇋	*IR*(*I*, *I*, *, *)	*k*_2_, *k*_-2_
*IR*(0, 0, 0, *)			⇋	*IR*(0, 0, *P*, *)	*k*_3_, *k*_-3_
*IR*(*I*, 0, 0, *)			⇋	*IR*(*I*, 0, *P*, *)	*k*_4_, *k*_-4_
*IR*(0, *I*, 0, *)			⇋	*IR*(0, *I*, *P*, *)	*k*_4_, *k*_-4_
*IR*(*I*, *I*, 0, *)			⇋	*IR*(*I*, *I*, *P*, *)	*k*_5_, *k*_-5_
*IR*(*, *, *P*, *)	+	*Shc*(*)	⇋	*IR*(*, *, *Shc*(*), *)	*k*_6_, *k*_-6_
*IR*(*I*, *I*, *Shc*(0), *)			⇋	*IR*(*I*, *I*, *Shc*(*P*), *)	*k*_7_, *k*_-7_
*IR*(*, *, *Shc*(*P*), *)	+	*Grb*2(*)	⇋	*IR*(*, *, *Grb*2(*), *)	*k*_8_, *k*_-8_
*IR*(*, *, *Grb*2(0), *)	+	*SOS*(0)	⇋	*IR*(*, *, *SOS*(0), *)	*k*_9_, *k*_-9_
*IR*(*, *, *Grb*2(0), *)	+	*SOS*(*P*)	⇋	*IR*(*, *, *SOS*(*P*), *)	*k*_10_, *k*_-10_
*IR*(*, *, *SOS*(0), *)			⇋	*IR*(*, *, *SOS*(*P*), *)	*k*_11_, *k*_-11_
*IR*(0, 0, *, 0)			⇋	*IR*(0, 0, *, *P*)	*k*_12_, *k*_-12_
*IR*(*I*, 0, *, 0)			⇋	*IR*(*I*, 0, *, *P*)	*k*_13_, *k*_-13_
*IR*(0, *I*, *, 0)			⇋	*IR*(0, *I*, *, *P*)	*k*_13_, *k*_-13_
*IR*(*I*, *I*, *, 0)			⇋	*IR*(*I*, *I*, *, *P*)	*k*_14_, *k*_-14_
*IR*(*, *, *, *P*)	+	*IRS*(*)	⇋	*IR*(*, *, *, *IRS*(*))	*k*_15_, *k*_-15_
*IR*(*I*, *I*, *, *IRS*(0))			⇋	*IR*(*I*, *I*, *, *IRS*(*P*))	*k*_16_, *k*_-16_
*IR*(*, *, *, *IRS*(*P*))	+	*Grb*2(*)	⇋	*IR*(*, *, *, *Grb*2(*))	*k*_17_, *k*_-17_
*IR*(*, *, *, *Grb*2(0))	+	*SOS*(0)	⇋	*IR*(*, *, *, *SOS*(0))	*k*_9_, *k*_-9_
*IR*(*, *, *, *Grb*2(0))	+	*SOS*(*P*)	⇋	*IR*(*, *, *, *SOS*(*P*))	*k*_10_, *k*_-10_
*IR*(*, *, *, *SOS*(0))			⇋	*IR*(*, *, *, *SOS*(*P*))	*k*_11_, *k*_-11_
*Shc*(0)			⇋	*Shc*(*P*)	*k*_18_, *k*_-18_
*Shc*(*P*)	+	*Grb*2(*)	⇋	*Shc*(*Grb*2(*))	*k*_8_, *k*_-8_
*Shc*(*Grb*2(0))	+	*SOS*(0)	⇋	*Shc*(*SOS*(0))	*k*_9_, *k*_-9_
*Shc*(*Grb*2(0))	+	*SOS*(*P*)	⇋	*Shc*(*SOS*(*P*))	*k*_10_, *k*_-10_
*Shc*(*SOS*(0))			⇋	*Shc*(*SOS*(*P*))	*k*_11_, *k*_-11_
*Grb*2(0)	+	*SOS*(0)	⇋	*Grb*2(*SOS*(0))	*k*_9_, *k*_-9_
*Grb*2(0)	+	*SOS*(*P*)	⇋	*Grb*2(*SOS*(*P*))	*k*_10_, *k*_-10_
*Grb*2(*SOS*(0))			⇋	*Grb*2(*SOS*(*P*))	*k*_11_, *k*_-11_
*SOS*(0)			⇋	*SOS*(*P*)	*k*_19_, *k*_-19_
*IRS*(0)			⇋	*IRS*(*P*)	*k*_20_, *k*_-20_
*IRS*(*P*)	+	*Grb*2(*)	⇋	*IRS*(*Grb*2(*))	*k*_17_, *k*_-17_
*IRS*(*Grb*2(0))	+	*SOS*(0)	⇋	*IRS*(*SOS*(0))	*k*_9_, *k*_-9_
*IRS*(*Grb*2(0))	+	*SOS*(*P*)	⇋	*IRS*(*SOS*(*P*))	*k*_10_, *k*_-10_
*IRS*(*SOS*(0))			⇋	*IRS*(*SOS*(*P*))	*k*_11_, *k*_-11_
*ER*(0, *, *)	+	*EGF *	⇋	*ER*(*E*, *, *)	*k*_21_, *k*_-21_
*ER*(*, 0, *)			⇋	*ER*(*, *P*, *)	*k*_22_, *k*_-22_
*ER*(*, *P*, *)	+	*Shc*(*)	⇋	*ER*(*, *Shc*(*), *)	*k*_23_, *k*_-23_
*ER*(*, *Shc*(0), *)			⇋	*ER*(*, *Shc*(*P*), *)	*k*_24_, *k*_-24_
*ER*(*, *Shc*(*P*), *)	+	*Grb*2(*)	⇋	*ER*(*, *Grb*2(*), *)	*k*_8_, *k*_-8_
*ER*(*, *Grb*2(0), *)	+	*SOS*(0)	⇋	*ER*(*, *SOS*(0), *)	*k*_9_, *k*_-9_
*ER*(*, *Grb*2(0), *)	+	*SOS*(*P*)	⇋	*ER*(*, *SOS*(*P*), *)	*k*_10_, *k*_-10_
*ER*(*, *SOS*(0), *)			⇋	*ER*(*, *SOS*(*P*), *)	*k*_11_, *k*_-11_
*ER*(*, *, 0)			⇋	*ER*(*, *, *P*)	*k*_25_, *k*_-25_
*ER*(*, *, *P*)	+	*Grb*2(*)	⇋	*ER*(*, *, Grb2(*))	*k*_26_, *k*_-26_
*ER*(*, *, *Grb*2(0))	+	*SOS*(0)	⇋	*ER*(*, *, *SOS*(0))	*k*_9_, *k*_-9_
*ER*(*, *, *Grb*2(0))	+	*SOS*(*P*)	⇋	*ER*(*, *, *SOS*(*P*))	*k*_10_, *k*_-10_
*ER*(*, *, *SOS*(0))			⇋	*ER*(*, *, *SOS*(*P*))	*k*_11_, *k*_-11_
*ER*(*E*, *, *)	+	*ER*(0, *, *)	⇋	*ER*_2_(*E*, *, *, 0, *, *)	*k*_27_, *k*_-27_
*ER*(0, *, *)	+	*ER*(0, *, *)	⇋	*ER*_2_(0, *, *, 0, *, *)	*k*_28_, *k*_-28_
*ER*(*E*, *, *)	+	*ER*(*E*, *, *)	⇋	*ER*_2_(*E*, *, *, *E*, *, *)	*k*_29_, *k*_-29_
*ER*_2_(0, *, *, *, *, *)	+	*EGF *	⇋	*ER*_2_(*E*, *, *, *, *, *)	*k*_30_, *k*_-30_
*ER*_2_(*, 0, *, *, *, *)			⇋	*ER*_2_(*, *P*, *, *, *, *)	*k*_31_, *k*_-31_
*ER*_2_(*, *Shc*(0), *, *, *, *)			⇋	*ER*_2_(*, *Shc*(*P*), *, *, *, *)	*k*_32_, *k*_-32_
*ER*_2_(*, *P*, *, *, *, *)	+	*Shc*(*)	⇋	*ER*_2_(*, *Shc*(*), *, *, *, *)	*k*_23_, *k*_-23_
*ER*_2_(*, *Shc*(*P*), *, *, *, *)	+	*Grb*2(*)	⇋	*ER*_2_(*, *Grb*2(*), *, *, *, *)	*k*_8_, *k*_-8_
*ER*_2_(*, *Grb*2(0), *, *, *, *)	+	*SOS*(0)	⇋	*ER*_2_(*, *SOS*(0), *, *, *, *)	*k*_9_, *k*_-9_
*ER*_2_(*, *Grb*2(0), *, *, *, *)	+	*SOS*(*P*)	⇋	*ER*_2_(*, *SOS*(*P*), *, *, *, *)	*k*_10_, *k*_-10_
*ER*_2_(*, *SOS*(0), *, *, *, *)			⇋	*ER*_2_(*, *SOS*(*P*), *, *, *, *)	*k*_11_, *k*_-11_
*ER*_2_(*, *, 0, *, *, *)			⇋	*ER*_2_(*, *, 0, *, *, *)	*k*_33_, *k*_-33_
*ER*_2_(*, *, *P*, *, *, *)	+	*Grb*2(*)	⇋	*ER*_2_(*, *, *Grb*2(*), *, *, *)	*k*_34_, *k*_-34_
*ER*_2_(*, *, *Grb*2(0), *, *, *)	+	*SOS*(0)	⇋	*ER*_2_(*, *, *SOS*(0), *, *, *)	*k*_9_, *k*_-9_
*ER*_2_(*, *, *Grb*2(0), *, *, *)	+	*SOS*(*P*)	⇋	*ER*_2_(*, *, *SOS*(*P*), *, *, *)	*k*_10_, *k*_-10_
*ER*_2_(*, *SOS*(0), *, *, *, *)			⇋	*ER*_2_(*, *, *SOS*(*P*), *, *, *)	*k*_11_, *k*_-11_

#### Complete mechanistic model vs. exactly reduced model

A complete mechanistic model of the described network of EGF and insulin receptor crosstalk comprises 42,956 reactions and 5,182 ODEs. According to the assumed process interactions the complete network can be parametrized by 68 kinetic parameters which can be seen in Table 7. The exact numerical value of these parameters does not play an important role in this context. The main purpose of this model is to illustrate the possibilities offered by the new reduction methods. Hence, the model equations are normalized to relative concentrations. The overall concentration of the considered components EGFR, IR, Shc, Grb2, SOS and IRS are set to 100 percent. The kinetic parameters are chosen such that the model qualitatively shows the expected behavior. We will focus on time plots of the quantities [*IR*(*, *, *SOS*(*), *)], [*IR*(*, *, *, *SOS*(*))], [*EGFR*(*, *SOS*(*), *).*] and [*EGFR*(*, *, *SOS*(*)).*].

The complete mechanistic model can be generated by BIONETGEN or other similar rule-based modeling tools. This example was modeled using the software tool ALC [29]. ALC allows the generation of combinatorial network models and produces output files for both MATLAB and MATHEMATICA. One simulation run with the MATLAB integrator ode15s took several hours using an Intel^® ^Xeon™ CPU with 3.06 GHz and 2 GB main memory. The simulation can be sped up by providing an analytically derived Jacobian matrix of the ODE system. All non-zero elements of the Jacobian matrix have been analytically calculated using MATHEMATICA and afterwards have been exported to MATLAB. The resulting simulation files have a size of over 13 MB, and a single simulation run still takes about half an hour. All files required to simulate the complete reaction network are provided in a ZIP-files [see Additional file 1].

**Figure 5:**
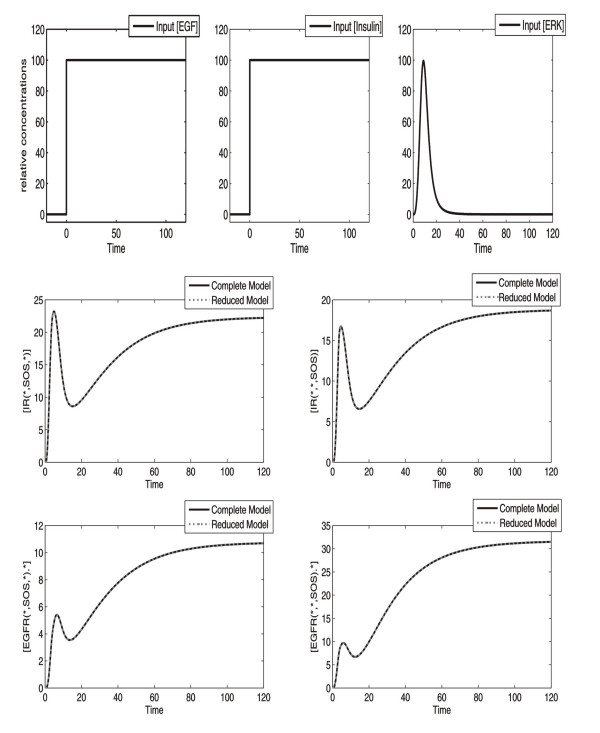
Simulation results of the generated crosstalk model. The kinetic parameters of the model have been chosen such that the system qualitatively shows the expected behavior. All quantities are depicted in relative concentrations. The overall concentrations of all involved components have been set to 100. The displayed curves show the chosen input signals [*EGF*], [*insulin*] and [*ERK*] as well as the output concentrations [*IR*(*, *SOS*, *)], [*IR*(*, *, *SOS*)], [*EGFR*(*, *SOS*, *).*] and [*EGFR*(*, *, *SOS*).*].

An exactly reduced version of the crosstalk model was generated using the reduced order modeling approach we introduced above. The definition of molecules, processes and process interactions (steps 1–3 of the method) is already given in the previous *Model definition *section. The process interaction graph corresponds to the arrows drawn in Figure 4. In order to get comparable results for all occurring binding and phosphorylation processes each of them was chosen as output process (step 4). The process interaction graph of the considered system can be dissected into four subgraphs (step 5). Until now this step has not been automatized but an automatization would be possible. Each subgraph describes one intracellular binding domain either of the EGF or the insulin receptor. However, due to the multifunctionality of the Grb2 binding domain all four subgraphs comprise the Grb2-SOS binding process as well as the serine/threonine phosphorylation of SOS. Consequently, the four subgraphs have to be simultaneously modeled and all species have to be simulantiously balanced. We use the modeling tool ALC to model the four submodels [29]. The input file with which ALC generates the ODEs is provided as additional file [see Additional file 2]. A link to a downloadable version of ALC can be found in Koschorreck *et al*. [29]. The resulting model comprises 1,826 reactions and 391 ODEs which already is a significant reduction compared to the complete model. A further reduction can be achieved by transforming the model to the previously introduced occurrence levels and subsequent elimination of redundant, unobservable and uncontrollable system dynamics (steps 7 and 8). These steps have been performed using MATHEMATICA. The MATHEMATICA code can also be found in the Additional files section [see Additional file 3]. The final and exactly reduced model of the network consists of 87 ODEs, which can be divided into six unidirectionally coupled modules. One of these modules, which consists of four ODEs, describes EGF binding and EGFR homodimerization. Another module specifies insulin binding to the insulin receptor and comprises two ODEs. Six ODEs are required to model IR phosphorylation at the IRS domain and subsequent IRS binding. Shc binding to EGFR as well as IR and the related domain phosphorylations form another module with a total number of 18 ODEs. The largest module consists of 32 ODEs and describes Grb2 binding to the EGF receptor as well as to phosphorylated Shc. The last module comprises all variables describing SOS binding and SOS phosphorylation and consists of 25 ODEs. One simulation run of this exactly reduced model only takes a few seconds. The size of the simulation file is 37.4 KB [see Additional file 4]. In Figure 5 it is shown that both models also provide exactly the same results for the considered output variables.

## Conclusion

Mathematical models of biochemical reaction networks play an increasing role in cytological research. Most of the underlying reaction networks are far too complex to facilitate an intuitive understanding. In this contribution, the focus is on ODE based dynamic modeling of receptor mediated signal transduction in mammalian cells like insulin or epidermal growth factor (EGF) signaling. These networks share some common features. Ligand binding to a receptor triggers conformational changes that facilitate receptor dimerization and/or phosphorylation of numerous residues. The subsequent formation of multiprotein signaling complexes on these receptors and their scaffolding adaptor proteins initiates a variety of signaling pathways. The main problem that occurs in modeling these networks using common modeling strategies is the enormous number of feasible multiprotein species and the high complexity of the related reaction networks. The main contribution of this work for ODE based modeling of signal transduction pathways is the extension and further development of an existing model reduction technique and the introduction of a reduced order modeling technique that allows to generate manageable reduced models accounting for the dynamic effects of combinatorial complexity.

For common in- and output signals the number of unobservable and uncontrollable model states depends on the occurring process interactions and is usually fairly high for a complete mechanistic model. The elimination of uncontrollable and unobservable state variables can be achieved by a linear and hierarchically structured state space transformation, which additionally facilitate a modularization of the model equations. Due to the enormous size of many real signaling cascades the generation of a complete mechanistic model and its subsequent reduction is not practical. An alternative approach is directly based on the process interaction pattern of the regarded system. All occurring process interactions can be integrated in an interaction graph which is subsequently dissected into independent interaction subgraphs. This exact reduced order modeling technique is used to generate a reduced model of EGF and insulin receptor crosstalk. This method allows to fairly reduce the complete mechanistic model with 5,182 ODEs to solely 87. Simulation studies show that the reduced model has exactly he same input/output behavior than the complete mechanistic model.

Thus, the results of this contribution provide new and powerful tools for dynamic modeling of combinatorial reaction networks like they occur in signal transduction. The introduced reduction techniques facilitate the generation of fairly reduced and modularized dynamic models. The modular structure of the resulting models also reduces the complexity of parameter estimation. Furthermore, the availability of an alternative reduced order modeling approach also facilitates the handling of very large and complex signaling networks. This property is of immense practical relevance since most real signaling cascades are too complex to be translated into a complete mechanistic model which is subsequently reduced.

## Authors' contributions

HC and DF developed the mathematical methods. HC designed and analyzed the discussed examples. EDG initiated, supervised and coordinated the project. All authors wrote the manuscript and approved the final version.

## Supplementary Material

Additional file 1 Matlab simulation files for the complete 5,182 ODE model of EGF and insulin receptor crosstalk. Click here for file

Additional file 2 ALC input file to generate an exactly reduced version of the EGF and insulin receptor model. Click here for file

Additional file 3 Transformation of the ALC generated model according to the proposed approach and subsequent elimination of redundant information. Click here for file

Additional file 4 Matlab simulation file for the reduced ODE model of EGF and insulin receptor crosstalk. Click here for file
